# Urinary Microcholesterol and Adverse Kidney Outcomes in CKD

**DOI:** 10.1016/j.ekir.2026.106549

**Published:** 2026-04-21

**Authors:** Hirotaka Furuta, Ryosuke Umeda, Masato Hoshi, Nanaka Morita, Fumiaki Sato, Hiroyuki Yokoi, Shun Minatoguchi, Soshiro Ogata, Kazuo Takahashi, Shigehisa Koide, Hiroyuki Naruse, Midori Hasegawa, Yukio Yuzawa, Hiroki Hayashi, Naotake Tsuboi

**Affiliations:** 1 Department of Nephrology, Fujita Health University School of Medicine, 1-98, Kutsukakecho Dengakugakubo, Toyoake city, Aichi, Japan; 2 Department of Disease Systems Analysis Medicine, Fujita Health University School of Health Sciences, Kutsukakecho Dengakugakubo 1-98, Toyoake city, Aichi, Japan; 3 Department of Medical Science Technology, School of Health Science at Narita, International University of Health and Welfare, 4-3 Kozunomori, Narita 286-8686, Japan; 4 Department of Biomedical Molecular Sciences, Fujita Health University School of Medicine, 1-98, Kutsukakecho Dengakugakubo, Toyoake city, Aichi, Japan; 5 Department of Clinical Pathophysiology, Fujita Health University School of Health Sciences, Kutsukakecho Dengakugakubo 1-98, Toyoake city, Aichi, Japan; 6 Faculty of Nursing, Fujita Health University School of Health Science, Kutsukakecho Dengakugakubo 1-98, Toyoake city, Aichi, Japan

**Keywords:** chronic kidney disease, lipids, proteinuria

## Abstract

**Introduction:**

Urinary microcholesterol (U-mCHO) may reflect tubular or glomerular injury through impaired lipid handling; nonetheless, its prognostic value in chronic kidney disease (CKD) remains unclear. We examined whether U-mCHO is associated with adverse kidney outcomes.

**Methods:**

We conducted a cohort study of patients with CKD (baseline eGFR ≥ 15 ml/min per 1.73 m^2^), among whom baseline U-mCHO was measured between April 2022 and July 2022 and who were followed until December 2025. The primary outcome was major adverse kidney events (MAKE) with a 50% estimated glomerular filtration rate (eGFR) decline (MAKE50), defined as a composite of a ≥ 50% reduction in eGFR, initiation of kidney replacement therapy (KRT), or kidney-related death. Associations were evaluated using multivariable Cox models. Subgroup analyses focused on patients with low proteinuria (urinary protein-to-creatinine ratio [UPCR] < 0.5 g/g) using an alternative composite outcome (MAKE30) based on a 30% decline in eGFR.

**Results:**

A total of 1562 patients were included. The mean U-mCHO level was 3.0 ± 5.8 mg/g creatinine. Higher U-mCHO levels were associated with greater MAKE50 risk (adjusted hazard ratio [HR] for highest vs. lowest quartile: 7.30; 95% confidence interval [CI]: 2.74–19.49). This association was consistent when U-mCHO was modeled as a log-transformed continuous variable. The association remained consistent across subgroups and persisted in patients with low proteinuria (HR for MAKE30: 3.06; 95% CI: 1.37–6.85), with no interaction by proteinuria level (*P* = 0.89).

**Conclusion:**

U-mCHO is independently associated with MAKE50 in CKD. The association with MAKE30 was also observed in patients with low proteinuria, supporting U-mCHO as a potential noninvasive biomarker of kidney lipid injury.

## Graphical abstract

**Figure:**
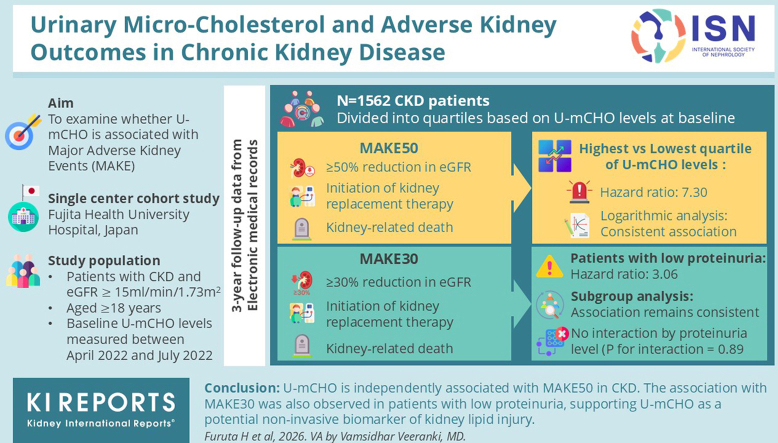

The primary route of cholesterol elimination is via hepatic conversion to bile acids, which are secreted into the small intestine and then reabsorbed and transported back to the liver by a process called enterohepatic circulation. Approximately 5% of cholesterol is ultimately excreted in feces, whereas trace amounts are also reported to be excreted in the urine.^1^^,^^2^ In the proximal tubule, the endocytic receptors megalin and cubilin mediate the reabsorption of various ligands, including lipid-related molecules such as filtered high-density lipoprotein (HDL) and its main apolipoprotein component, apolipoprotein A-I.^3^, ^4^, ^5^ Lipiduria is particularly prominent in patients with nephrotic syndrome.^6^ In these patients, lipid components can be detected in the urine, including cholesterol, triglycerides, free fatty acids, and phospholipids. These observations suggest that, under physiological conditions, a small amount of cholesterol and other lipid-related molecules is continuously filtered and subsequently reclaimed by the proximal tubule. Lipiduria may become apparent when this balance is disrupted, either due to increased glomerular permeability or impaired tubular reabsorption.

Urinary lipids may exhibit biological activity that contributes to kidney injury. Filtered HDL, the predominant urinary lipoprotein in nephrotic states, has been shown to stimulate endothelin-1 production in human proximal tubular cells, suggesting a role for urinary lipids in promoting tubulointerstitial injury.^7^ Furthermore, experimental studies have shown that albumin-bound free fatty acids in the urine can directly cause severe proximal tubular injury in mouse models.^8^ These findings indicate that urinary lipids may contribute to kidney injury through multiple mechanisms, beyond serving as mere markers of glomerular damage.

Building on these mechanistic insights, recent studies have gradually reported associations between lipiduria and CKD using lipidomics approaches. Cross-sectional studies in both diabetic and nondiabetic CKD have demonstrated these associations. In diabetic nephropathy, lysophosphatidic acid and lysophosphatidylcholine were elevated and increased with higher albuminuria,^9^ whereas in nondiabetic CKD, sphingomyelins and phosphatidylcholines were among the metabolites linked to disease status.^10^ In contrast, in a comprehensive meta-analysis of candidate biomarkers for CKD progression, lipid-related markers were notably absent, underscoring that urinary lipids had long been overlooked in prognostic research.^11^ This view has recently shifted— large-scale prospective metabolomics in CKD demonstrated that several lipid-related urinary metabolites, such as phosphatidylcholines and sphingomyelins, were significantly associated with kidney failure and mortality in time-to-event analyses.^12^ However, urinary cholesterol itself was not captured in these metabolomic approaches.^2^^,^^10^^,^^13^ In this context, Hotta *et al.*^14^ uniquely proposed that urinary microcholesterol (U-mCHO), measured using a high-sensitivity enzymatic cycling assay, reflects glomerular injury and may help distinguish progressive glomerular diseases; however, interpretation of their findings is tempered by small sample size, restriction to glomerular diseases, and dichotomous outcome assessment without time-to-event analyses.

To address these gaps, we conducted a longitudinal cohort study in a real-world CKD outpatient population to examine whether U-mCHO is associated with MAKE, and we also performed cross-sectional analyses to characterize the distribution and clinical correlates of U-mCHO at baseline. As a secondary, exploratory aim, we examined whether U-mCHO provides incremental predictive information beyond conventional clinical risk factors.

## Methods

### Patients

We conducted a cohort study of consecutive CKD outpatients at Fujita Health University Hospital. Baseline urinary mCHO levels were measured between April 2022 and July 2022, and subsequent outcomes were ascertained from electronic medical records through December 2025. Patients were eligible if they had a diagnosis of CKD, defined as abnormalities of kidney structure or function persisting for ≥ 3 months, identified by reduced eGFR (<60 ml/min per 1.73 m^2^) and/or evidence of kidney damage (e.g., persistent proteinuria, urinary sediment abnormalities, imaging, or histology) and were aged ≥ 18 years. We excluded patients with baseline eGFR <15 ml/min per 1.73 m^2^ (given the risk of imminent KRT and potential influence on short-term composite outcomes), those already receiving KRT, those with acute kidney injury or acute kidney disease, or those lacking baseline serum creatinine data. This study was conducted in accordance with the Declaration of Helsinki and was approved by the ethics review board of Fujita Health University (No. HM25-381 and HM25-382). Written informed consent was waived because of the noninterventional study design, and an opt-out procedure was implemented on the hospital website. All data were anonymized before analysis to protect patient confidentiality.

### Measurement of Urinary Microcholesterol

Urinary mCHO levels were measured using a high-sensitivity enzymatic cycling method, in which cholesterol undergoes reversible oxidation in the presence of thio- nicotinamide adenine dinucleotide and reduced nicotinamide adenine dinucleotide.^15^ This redox cycling reaction is repeated multiple times to amplify the signal, and the resulting generation of thio-reduced nicotinamide adenine dinucleotide is quantified by monitoring the rate of absorbance increase. Measurements were performed using an automatic analyzer 3500 (Hitachi High-Tech Corporation, Tokyo, Japan) with a dual-wavelength setting of 405/505 nm, based on urine samples collected during the study period.

### Clinical Evaluation

Clinical data at baseline (the time of U-mCHO measurement) were collected, including age, sex (defined as assigned at birth), body mass index (BMI), mean arterial pressure, smoking status, presence of diabetes mellitus, medication use, UPCR, estimated glomerular filtration rate (eGFR), serum creatinine, serum albumin, triglycerides, total cholesterol, low-density lipoprotein (LDL) cholesterol, and HDL cholesterol. Underlying kidney diseases were determined based on the diagnoses and clinical courses documented in the medical records. In addition, longitudinal eGFR value was extracted through December 2025 for outcome ascertainment. eGFR was calculated using the equation for the Japanese population.^16^

### Outcome

The primary outcome (MAKE50) was defined as the time from the date of U-mCHO measurement (time 0) to the earliest occurrence of a ≥ 50% decline in eGFR, initiation of KRT, or kidney-related death. A ≥ 50% eGFR decline was defined as the first observed eGFR value ≤ 50% of baseline and was confirmed on a subsequent measurement ≥ 28 days later. If no measurement was available ≥ 28 days after the initial decline, the participant was not classified as having a confirmed decline (censored at the last eGFR measurement). Each component of MAKE was also evaluated separately as secondary outcomes. In the component-specific analyses, initiation of KRT before confirmation of the ≥ 50% eGFR decline was treated as a censoring event for the eGFR-decline end point. Follow-up was terminated at the time of outcome occurrence; otherwise, participants were censored at nonkidney-related death, transfer to another hospital, or the last available follow-up. Patients typically underwent blood testing at each visit. For subgroup analyses, we additionally applied an alternative composite end point defined by a ≥ 30% decline in eGFR, in place of the 50% threshold (MAKE30), to increase the number of outcome events and improve statistical stability within subgroups.

### Statistical Analysis

Baseline characteristics were summarized as mean (SD) for continuous variables and number (percentage) for categorical variables.

To evaluate the association between U-mCHO levels and kidney outcomes, Kaplan–Meier survival curves with log-rank tests were generated for MAKE50 and for each of its individual components as secondary outcomes. As a sensitivity analysis for competing risks, we additionally performed cumulative incidence function analyses and Fine–Gray subdistribution hazard models (Supplementary Materials).

For multivariable analysis, Cox proportional hazards models were constructed using 3 approaches. First, U-mCHO was categorized into quartiles and baseline proteinuria was categorized into 3 groups (UPCR <0.15, 0.15–0.5, and ≥ 0.5 g/g). Quartiles of U-mCHO were referred to as Q1 (lowest) to Q4 (highest). Second, we assessed the linear trend across the U-mCHO quartiles using *P* for trend. Third, U-mCHO was treated as a continuous, log-transformed variable (log10). All 3 approaches were applied across 3 sequentially adjusted models as follows: Model 1 included age, sex, baseline eGFR, and UPCR; Model 2 additionally included mean arterial pressure, LDL cholesterol, and diabetic kidney disease (DKD) status; and Model 3 was further adjusted for the use of renin-angiotensin system (RAS) inhibitors, statins, and sodium-glucose cotransporter 2 (SGLT2) inhibitors. Adjustment covariates were prespecified *a priori* based on clinical relevance and potential confounding (rather than data-driven selection). We assessed the functional form of continuous variables using martingale residual plots with locally weighted scatterplot smoothing and modeled them as linear terms. The proportional hazards assumption was evaluated using Schoenfeld residuals. When nonproportionality was suggested for U-mCHO, we performed sensitivity analyses using a time-dependent Cox model with a covariate-by-time interaction and landmark analyses (365 and 730 days).

In subgroup analyses, effect modification was assessed across age, sex, BMI, eGFR, LDL cholesterol, DKD status, and medication use (RAS inhibitors, SGLT2 inhibitors, and statins) using Model 3, with MAKE30 as the exploratory outcome. Effect modification was tested in the overall cohort by fitting Model 3 with a multiplicative interaction term between log-transformed U-mCHO and each subgroup indicator; subgroup-specific HR were derived from the main and interaction coefficients. This interaction-based approach was also applied to prespecified subgroups defined by cause of kidney disease and by glomerular disease status (glomerular vs. nonglomerular). When subgroup definitions were derived from clinical classification variables overlapping with DKD status, DKD was not included as an adjustment covariate to avoid collinearity/overadjustment. For baseline proteinuria, we instead performed UPCR-stratified Cox models (UPCR <0.5 vs. ≥ 0.5 g/g), and tested effect modification in the overall cohort by adding a log-transformed U-mCHO × UPCR stratum interaction term to Model 3 (excluding UPCR from covariates). Subgroups were prespecified and defined as follows: age (< 60 vs. ≥ 60 years), BMI (<25 vs. ≥ 25 kg/m^2^), eGFR (< 45 vs. ≥ 45 ml/min per 1.73 m^2^), and LDL cholesterol (<120 vs. ≥ 120 mg/dl).

As an exploratory, hypothesis-generating analysis (reported in the Supplementary Materials), time-dependent receiver operating characteristic analysis using inverse probability of censoring weighting was performed based on Model 3, comparing models with and without U-mCHO. To reduce optimism, discrimination, and prediction error were evaluated using out-of-fold predictions from 10-fold cross-validation. We additionally evaluated time-dependent prediction error using the inverse probability of censoring weighting Brier score (prediction error curves). Prediction performance was assessed at 1, 2, and 3 years using 2000 bootstrap resamples.

To examine the distribution of U-mCHO, we created histograms of its untransformed values. Independent associations were further assessed using multivariable linear regression, in which log-transformed U-mCHO was used as the dependent variable and covariates were aligned with those in Model 3. Regression coefficients were expressed as percent changes per unit increase in each covariate, along with the corresponding *P* values. As supplementary analyses, we also included scatter plots of log-transformed U-mCHO against 8 clinical variables, as well as a density heatmap showing the average log-transformed U-mCHO by UPCR and eGFR levels.

The significance tests were 2-sided, and the significance level for all analyses was *P* < 0.05. Missing data for the analytic variables were handled using complete case analysis (12% excluded). All statistical analyses were conducted using Python (version 3.13.5, Python Software Foundation, Wilmington, DL). Key packages included pandas 2.3.0, NumPy 2.3.0, SciPy 1.15.3, lifelines 0.30.0, scikit-learn 1.7.2, and Matplotlib 3.10.3 (versions recorded at the time of analysis).Only the time-varying coefficient Cox model for assessing nonproportional hazards was fitted in R (version 4.3.1, Posit PBC, Boston, MA) using the survival package (version 3.5.5).

## Results

### Baseline Clinical Characteristics of the Study Population

**Figure 1:**
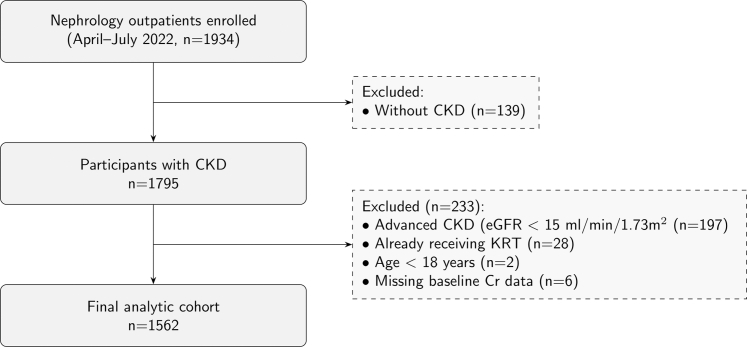
Patient flow diagram of the study cohort. A total of 1934 nephrology outpatients were enrolled between April and July 2022 and were prospectively followed until December 2025. After excluding 139 patients without chronic kidney disease (CKD) and an additional 233 patients (197 with advanced CKD (eGFR < 15 ml/min per 1.73 m^2^), 28 already receiving KRT, 6 with missing baseline creatinine (Cr) data, and 2 younger than 18 years), the final analytic cohort comprised 1,562 patients. Cr, creatinine; eGFR, estimated glomerular filtration rate; KRT, kidney replacement therapy

Of the 1934 nephrology outpatients enrolled between April 2022 and July 2022, 139 without CKD were excluded, leaving 1795 participants with CKD. An additional 233 patients were excluded because they were advanced CKD (eGFR < 15 ml/min per 1.73 m^2^) (n = 197), already receiving KRT (n = 28), were < 18 years (*n* = 2), or lacked baseline serum creatinine data (n = 6). The final analytic cohort thus comprised 1562 patients (Figure 1). This cohort served as the overall analytic cohort for subsequent analyses. Baseline data are summarized in Table 1. The mean age was 64 ± 17 years, and 45% were female. Patients in Q4 U-mCHO were older (67 ± 18 years) than those in Q1 U-mCHO (62 ± 17 years). The mean BMI was 23.6 ± 4.2 kg/m^2^, and the mean arterial pressure was 91.9 ± 12.3 mm Hg. Current smoking was reported in 10% of participants, whereas 32% were former smokers and 50% had never smoked. Diabetes mellitus was more prevalent in Q4 U-mCHO (38%) than in Q1 U-mCHO (23%). In terms of clinical diagnosis, 15% of patients were diagnosed with DKD, 16% with nephrosclerosis, 38% with glomerulonephritis, and 30% with other etiologies. The prevalence of DKD was also higher in Q4 U-mCHO (26%) compared with Q1 U-mCHO (13%).

**Table 1:** Baseline characteristics of the study cohort according to U-mCHO quartiles

Variable	Subcategory	Overall	Q1 U-mCHO	Q2 U-mCHO	Q3 U-mCHO	Q4 U-mCHO
Number		1562	391	390	390	391
Age		64 (17)	62 (17)	63 (17)	64 (17)	67 (18)
Sex, female		704 (45%)	97 (25%)	176 (45%)	211 (54%)	220 (56%)
BMI		23.6 (4.2)	23.7 (3.9)	23.6 (4.3)	23.5 (4.1)	23.7 (4.5)
Smoking	Current	151 (10%)	36 (9%)	45 (12%)	37 (9%)	33 (8%)
	Never	788 (50%)	172 (44%)	185 (47%)	209 (54%)	222 (57%)
	Past	495 (32%)	154 (39%)	122 (31%)	110 (28%)	109 (28%)
Mean arterial pressure, mm Hg		91.9 (12.3)	91.5 (11.9)	91.5 (11.7)	91.2 (11.6)	93.5 (13.7)
Albumin, g/dl		3.9 (0.4)	4.1 (0.3)	4.0 (0.3)	3.9 (0.4)	3.7 (0.5)
BUN, mg/dl		24.1 (12.1)	22.4 (9.9)	23.2 (11.5)	23.0 (11.6)	28.0 (14.1)
eGFR, ml/min per 1.73 m^2^		47 (24)	48 (23)	50 (24)	51 (24)	41 (25)
CKD stage	G1	100 (6%)	22 (6%)	29 (7%)	28 (7%)	21 (5%)
	G2	283 (18%)	65 (17%)	75 (19%)	93 (24%)	50 (13%)
	G3a	340 (22%)	105 (27%)	93 (24%)	80 (21%)	62 (16%)
	G3b	415 (27%)	115 (29%)	114 (29%)	98 (25%)	88 (23%)
	G4	424 (27%)	84 (21%)	79 (20%)	91 (23%)	170 (43%)
Total cholesterol, mg/dl		186 (39)	182 (35)	186 (39)	188 (39)	189 (44)
LDL cholesterol, mg/dl		99 (31)	100 (28)	98 (31)	100 (30)	99 (36)
HDL cholesterol, mg/dl		54 (17)	51 (16)	55 (18)	56 (17)	55 (17)
Triglyceride, mg/dl		143 (88)	144 (81)	136 (76)	144 (92)	147 (98)
UPCR, g/g		0.9 (1.6)	0.2 (0.3)	0.4 (0.4)	0.6 (0.8)	2.3 (2.5)
Urinary mCHO, mg/g		3.0 (5.8)	0.7 (0.2)	1.2 (0.1)	1.9 (0.3)	8.2 (9.8)
Cause of kidney disease	Diabetic kidney disease	232 (15%)	49 (13%)	38 (10%)	45 (12%)	100 (26%)
	Nephrosclerosis	254 (16%)	84 (21%)	61 (16%)	60 (15%)	49 (13%)
	Glomerulonephritis	600 (38%)	141 (36%)	151 (39%)	170 (44%)	138 (35%)
	Others	476 (30%)	117 (30%)	140 (36%)	115 (29%)	104 (27%)


Variable		Overall	Q1 U-mCHO	Q2 U-mCHO	Q3 U-mCHO	Q4 U-mCHO
Diabetes mellitus		432 (28%)	88 (23%)	95 (24%)	101 (26%)	148 (38%)
Drugs						
Antihypertensive agents		1307 (84%)	313 (80%)	314 (81%)	329 (84%)	351 (90%)
RAS inhibitor		1062 (68%)	250 (64%)	260 (67%)	261 (67%)	291 (74%)
ARNI		163 (10%)	33 (8%)	30 (8%)	51 (13%)	49 (13%)
SGLT2 inhibitor		454 (29%)	107 (27%)	108 (28%)	107 (27%)	132 (34%)
GLP-1 agonist		65 (4%)	13 (3%)	11 (3%)	9 (2%)	32 (8%)
Lipid-lowering agents		939 (60%)	227 (58%)	225 (58%)	229 (59%)	258 (66%)
Statin		792 (51%)	190 (49%)	190 (49%)	188 (48%)	224 (57%)
Fibrate		18 (1%)	6 (2%)	3 (0.8%)	7 (2%)	2 (0.5%)
PPARα modulator		49 (3%)	18 (5%)	6 (2%)	13 (3%)	12 (3%)
Cholesterol absorption inhibitor		153 (10%)	32 (8%)	42 (11%)	34 (9%)	45 (12%)
PCSK9 inhibitor		7 (0.4%)	2 (0.5%)	1 (0.3%)	2 (0.5%)	2 (0.5%)
Omega-3 fatty acids		209 (13%)	55 (14%)	48 (12%)	52 (13%)	54 (14%)
Probucol		2 (0.1%)	1 (0.3%)	0 (0.0%)	0 (0.0%)	1 (0.3%)
Nicotinic acid		36 (2%)	8 (2%)	6 (2%)	11 (3%)	11 (3%)
Resin		7 (0.4%)	2 (0.5%)	1 (0.3%)	3 (0.8%)	1 (0.3%)

ARNI, angiotensin receptor–neprilysin inhibitor; BMI, body mass index; BUN, blood urea nitrogen; CKD, chronic kidney disease; eGFR, estimated glomerular filtration rate; GLP-1, glucagon-like peptide-1; PCSK9, proprotein convertase subtilisin/kexin type 9; PPAR, peroxisome proliferator–activated receptor; RAS, renin–angiotensin system; SGLT2, sodium–glucose cotransporter 2; UPCR, urine protein-to-creatinine ratio; U-mCHO, urinary microcholesterol.

Values are presented as mean ± SD or *n* (%).

The mean U-mCHO level was 3.0 ± 5.8 mg/g, and the mean UPCR was 0.9 ± 1.6 g/g, rising progressively from 0.2 g/g in Q1 U-mCHO to 2.3 g/g in Q4 U-mCHO. The mean eGFR was 47 ± 24 ml/min per 1.73 m^2^. The CKD stage distribution was G1 6% (n = 100), G2 18% (n = 283), G3a 22% (n = 340), G3b 27% (n = 415), and G4 27% (n = 424), with a higher proportion of stage G4 in Q4 U-mCHO (43%) than in Q1 U-mCHO (21%). As for serum lipids, the mean triglyceride level was 143 ± 88 mg/dl, total cholesterol was 186 ± 39 mg/dl, LDL cholesterol was 99 ± 31 mg/dl, and HDL cholesterol was 54 ± 17 mg/dl. These values were generally comparable across the U-mCHO quartiles. Regarding medication use, 84% were prescribed antihypertensive drugs, including RAS inhibitors (68%). Lipid-lowering agents were used in 60% of patients, most commonly statins (51%). SGLT2 inhibitors were used in 29% of patients.

### U-mCHO and Kidney Prognosis

**Figure 2:**
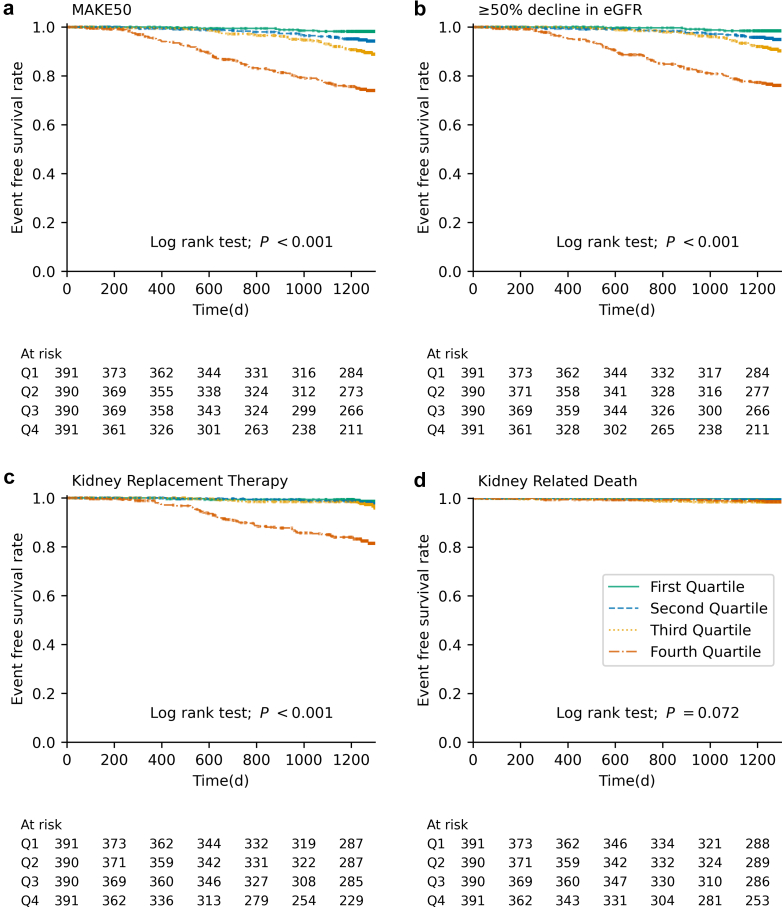
Kaplan–Meier curves for MAKE50 and its individual components according to U-mCHO quartiles. Kaplan–Meier curves showing cumulative event-free survival for kidney outcomes according to urinary microcholesterol (U-mCHO) quartiles. (a) Composite kidney outcome (MAKE50), defined as a ≥ 50% decline in estimated glomerular filtration rate (eGFR), initiation of KRT, or kidney-related death. (b) ≥ 50% decline in eGFR. (c) Initiation of KRT. (d) Kidney-related death. The highest U-mCHO quartile exhibited the greatest risk of adverse kidney outcomes (log-rank *p* < 0.001 for panels a–c). No significant difference was observed among quartiles for kidney-related death (panel d). Numbers at risk are shown below each panel under the corresponding Kaplan–Meier curve. Analytic cohort: *n* = 1562. MAKE, major adverse kidney events; MAKE50, composite of a ≥ 50% decline in eGFR, initiation of KRT, or kidney-related death; KRT, kidney replacement therapy

During a median follow-up of 41.6 months (IQR: 33.6–42.8), 142 patients (9.1%) experienced the primary kidney composite outcome. Kaplan–Meier analysis showed a clear early separation of survival curves according to U-mCHO quartiles, with the highest quartile exhibiting the greatest risk of adverse kidney events (log-rank *P* < 0.001; Figure 2A). Regarding secondary outcomes, patients in the highest U-mCHO quartile had a significantly greater risk of a 50% decline in eGFR (log-rank *P* < 0.001; Figure 2B) and initiation of KRT (log-rank *P* < 0.001; Figure 2C), whereas no significant difference was observed among quartiles for kidney-related death (Figure 2D). The crude incidence rates of each outcome across U-mCHO quartiles are summarized in Table 2. The incidence of MAKE50 increased across quartiles, reaching 29.0 per 1000 person-years in Q3 U-mCHO and 81.3 per 1000 person-years in Q4 U-mCHO. A similar pattern was observed for the secondary end points of a ≥ 50% eGFR decline and KRT, whereas no clear difference was noted for kidney-related death. These incidence rates are unadjusted and are presented for descriptive purposes; therefore, they can reflect baseline risk imbalances across quartiles. During follow-up, 99 participants died from nonkidney-related causes (Supplementary Table S2B).

**Table 2:** Crude incidence rates of kidney outcomes according to U-mCHO quartiles

Outcome	U-mCHO Quartile	Number	Events	Person-years	Crude incidence rate per 1000 PY	95% CI
Primary end point						
MAKE 50	All patients	1562	142	4576.6	31	26.1–36.6
	Q1 U-mCHO	391	6	1195.6	5.0	1.83–10.9
	Q2 U-mCHO	390	18	1177.9	15.3	9.05–24.2
	Q3 U-mCHO	390	34	1170.4	29.0	20.1–40.6
	Q4 U-mCHO	391	84	1032.7	81.3	64.9–101
Secondary end point						
≥ 50% eGFR decline	Q1 U-mCHO	391	5	1196.6	4.2	1.35–9.75
	Q2 U-mCHO	390	16	1188.1	13.5	7.69–21.9
	Q3 U-mCHO	390	29	1173.6	24.7	16.5–35.5
	Q4 U-mCHO	391	76	1035.6	73.4	57.8–91.9
Initiation of KRT	Q1 U-mCHO	391	4	1199.1	3.3	0.90–8.54
	Q2 U-mCHO	390	6	1199.2	5.0	1.83–10.9
	Q3 U-mCHO	390	10	1188.8	8.4	4.03–15.5
	Q4 U-mCHO	391	58	1073.7	54.0	41–69.8
Kidney-related death	Q1 U-mCHO	391	0	1203.5	0.0	0–2.49
	Q2 U-mCHO	390	1	1202.5	0.8	0.011–4.63
	Q3 U-mCHO	390	5	1193.7	4.2	1.35–9.77
	Q4 U-mCHO	391	4	1127.3	3.6	0.96–9.08
Alternative end point						
MAKE 30	All patients	1562	293	4319.7	67.8	60.3–76.1
	Q1 U-mCHO	391	30	1167.1	25.7	17.3–36.7
	Q2 U-mCHO	390	52	1132.6	45.9	34.3–60.2
	Q3 U-mCHO	390	68	1112.3	61.1	47.5–77.5
	Q4 U-mCHO	391	143	907.7	158.0	133–186

eGFR, estimated glomerular filtration rate; PY, person-years; U-mCHO, urinary microcholesterol.

Crude incidence rates are expressed per 1,000 person-years. Major adverse kidney events (MAKE) 50/30 were defined as a composite of a ≥50/30% decline in eGFR, initiation of KRT, or kidney-related death. Primary inference regarding the association is based on the multivariable Cox models in Table 3.

In Cox proportional hazards models, patients in higher quartiles of U-mCHO had an increased risk of the primary composite outcome compared with those in the lowest quartile, with the highest quartile showing the greatest risk (*P* for trend < 0.001; Table 3). Notably, the estimated HRs were already elevated in Q2 relative to Q1 and increased further across Q3 and Q4. Hazard ratios for U-mCHO quartiles were similar across Models 1 to 3 after sequential adjustment with additional clinically relevant covariates, suggesting that the association was robust to more extensive adjustment. When U-mCHO was treated as a log-transformed continuous variable, the results were consistent, with adjusted HRs ranging from 3.72 to 3.97 (per 1-unit increase in log-transformed U-mCHO, *P* < 0.001; Table 3). Higher UPCR categories and male sex were also independently associated with higher risk, whereas higher baseline eGFR was associated with lower risk. Statin use was not independently associated with the outcome. Full model outputs (β coefficients, HRs with 95% CIs, and P values) for Models 1 to 3 are provided in Supplementary Tables S3–S5.

**Table 3:** Hazard ratios for kidney outcomes according to U-mCHO levels

Outcome	Q1 U-mCHO	Q2 U-mCHO	Q3 U-mCHO	Q4 U-mCHO	*P* for trend (HR per 1-quartile increase)	*P*-value	Continuous	*P*-value
	HR (95% CI)	HR (95% CI)	HR (95% CI)	HR (95% CI)	HR (95% CI)		HR (95% CI)	
Primary end point (MAKE50)								
Model 1	1.0 (ref)	2.90 (1.06–7.94)	3.59 (1.32–9.72)	7.47 (2.81–19.83)	1.80 (1.44–2.26)	< 0.001	3.97 (2.64–5.97)	< 0.001
Model 2	1.0 (ref)	2.91 (1.06–7.98)	3.65 (1.34–9.90)	7.41 (2.78–19.75)	1.79 (1.42–2.25)	< 0.001	3.89 (2.57–5.90)	< 0.001
Model 3	1.0 (ref)	2.80 (1.02–7.70)	3.59 (1.32–9.75)	7.30 (2.74–19.49)	1.79 (1.42–2.25)	< 0.001	3.72 (2.45–5.65)	< 0.001
Secondary end point								
≥ 50% decline in eGFR								
Model 1	1.0 (ref)	2.11 (0.76–5.84)	2.24 (0.82–6.14)	5.02 (1.90–13.30)	1.69 (1.33–2.15)	< 0.001	3.51 (2.26–5.45)	< 0.001
Model 2	1.0 (ref)	2.13 (0.77–5.93)	2.28 (0.83–6.27)	4.88 (1.83–13.02)	1.66 (1.30–2.11)	< 0.001	3.69 (2.38–5.73)	< 0.001
Model 3	1.0 (ref)	2.04 (0.73–5.70)	2.26 (0.82–6.21)	4.76 (1.78–12.72)	1.65 (1.30–2.10)	< 0.001	3.84 (2.50–5.91)	< 0.001
Initiation of KRT								
Model 1	1.0 (ref)	1.36 (0.32–5.70)	1.44 (0.35–5.99)	7.23 (1.94–26.89)	2.52 (1.75–3.64)	< 0.001	6.24 (3.62–10.74)	< 0.001
Model 2	1.0 (ref)	1.40 (0.33–5.92)	1.51 (0.36–6.30)	7.32 (1.94–27.65)	2.47 (1.70–3.59)	< 0.001	5.96 (3.41–10.39)	< 0.001
Model 3	1.0 (ref)	1.38 (0.32–5.87)	1.51 (0.36–6.33)	7.13 (1.88–27.11)	2.44 (1.68–3.54)	< 0.001	5.88 (3.33–10.40)	< 0.001
Alternative end point (MAKE30)								
Model 1	1.0 (ref)	1.91 (1.17–3.12)	2.15 (1.31–3.52)	3.92 (2.41–6.37)	1.53 (1.33–1.76)	< 0.001	3.06 (2.26–4.15)	< 0.001
Model 2	1.0 (ref)	1.93 (1.18–3.16)	2.17 (1.32–3.57)	3.89 (2.38–6.34)	1.52 (1.32–1.75)	< 0.001	3.03 (2.23–4.13)	< 0.001
Model 3	1.0 (ref)	1.90 (1.16–3.11)	2.15 (1.31–3.53)	3.90 (2.39–6.35)	1.52 (1.32–1.76)	< 0.001	2.99 (2.19–4.08)	< 0.001

CI, confidence interval; DKD, diabetic kidney disease; eGFR, estimated glomerular filtration rate; HR, hazard ratio; KRT, kidney replacement therapy; MAKE, major adverse kidney events; NA, not applicable.

HRs and 95% CIs were derived from Cox proportional hazards models. Model 1 included age, sex, baseline eGFR, and UPCR; Model 2 additionally included mean arterial pressure, LDL cholesterol, and DKD status; and Model 3 further adjusted for the use of RAS inhibitors, statins, and SGLT2 inhibitors. Q1-U-mCHO served as the reference category. U-mCHO was also analyzed as a log-transformed continuous variable. MAKE50 was defined as a composite of a ≥50% decline in eGFR, initiation of KRT, or kidney-related death. P for trend was assessed by modeling U-mCHO quartiles as an ordinal variable (1–4); the reported HR represents the change in hazard per 1-quartile increase. Continuous HR is expressed per 1-unit increase in log10-transformed U-mCHO. Analytic cohort: n = 1373.

Sensitivity analyses were consistent with the primary findings. These included analyses using an alternative endpoint (MAKE30; ≥ 30% eGFR decline; Table 3, Supplementary Figure S1 and Table S1), competing-risk analyses using CIF and Fine–Gray models (Supplementary Figure S2 and Table S2 A), and additional model specifications (nonstatin lipid-lowering agents; Supplementary Table S6). Model diagnostics supported the functional form assumptions: martingale residual plots did not suggest marked nonlinearity for log-transformed U-mCHO or other continuous covariates (Supplementary Figure S3). Schoenfeld residual diagnostics suggested departure from the proportional hazards assumption for U-mCHO. In a time-varying coefficient Cox model including an interaction between log10(U-mCHO) and log(time/365), the interaction term was significant (Wald *P* = 0.01–0.03; likelihood ratio test *P* = 0.01–0.03 across Models 1–3), indicating attenuation of the association over follow-up; landmark analyses at 365 and 730 days showed a similar pattern (Supplementary Table S7 and Figure S4).

### Subgroup Analysis Among Patients With Low UPCR

**Figure 3:**
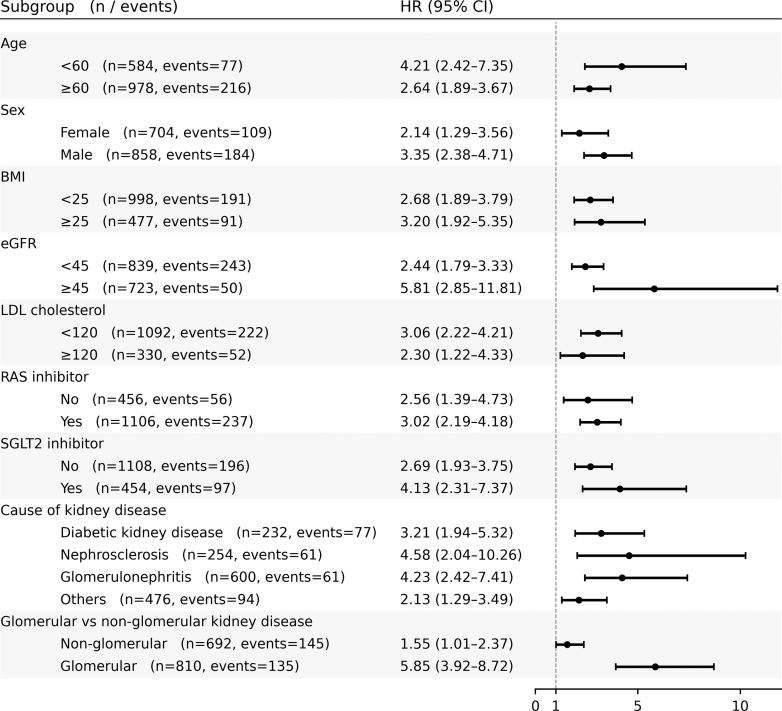
Subgroup analyses for MAKE30 according to U-mCHO levels. Subgroup analyses of the association between urinary microcholesterol (U-mCHO) and the risk of Major Adverse Kidney Events defined by a ≥ 30% decline in eGFR (MAKE30). HRs and 95% CIs for the risk of major adverse kidney events defined by MAKE30 are shown across subgroups. HRs were estimated using Cox proportional hazards models adjusted according to Model 3, with log-transformed U-mCHO as the predictor. Effect modification was assessed by including a multiplicative interaction term between log-transformed U-mCHO and each subgroup indicator. Associations between U-mCHO and MAKE30 were consistent across subgroups defined by age, sex, body mass index (BMI), baseline eGFR, LDL cholesterol, cause of kidney disease, anatomical site of kidney injury (glomerular vs. nonglomerular), and use of renin–angiotensin system (RAS) inhibitors, SGLT2 inhibitors, or statins. Analytic cohort: *n* = 1373. CI, confidence interval; eGFR, estimated glomerular filtration rate; HR, hazard ratio; LDL, low-density lipoprotein; MAKE, major adverse kidney events; RAS, renin–angiotensin system; SGLT2, sodium–glucose cotransporter 2; U-mCHO, urinary microcholesterol; UPCR, urine protein-to-creatinine ratio

We further focused on patients with low-level proteinuria, including 873 patients with a UPCR <0.5 g/g at baseline, representing a relatively low-proteinuria CKD population. In this subgroup, U-mCHO levels were generally lower, and baseline kidney function was slightly better than that in the overall cohort (Supplementary Table S8). In Cox models using MAKE30 as the outcome, log-transformed U-mCHO was associated with a higher risk of the outcome across all models (HR: 3.06; 95% CI: 1.37–6.85 in Model 3; Table 4). Effect modification by baseline proteinuria was assessed by testing the interaction between log-transformed U-mCHO and UPCR strata (UPCR < 0.5 vs. ≥ 0.5 g/g), and no significant interaction was observed (*P* for interaction = 0.89; Model 3). These findings suggest that U-mCHO provides prognostic information within a low-proteinuria CKD subgroup. In prespecified subgroup analyses across age, sex, BMI, eGFR, LDL cholesterol, cause of kidney disease, anatomical site of kidney injury, and medication use (RAS inhibitors, SGLT2 inhibitors, and statins), the association between U-mCHO and MAKE30 remained generally consistent, with elevated risks observed across most subgroups and no evidence of significant heterogeneity (Figure 3).

**Table 4:** Association of U-mCHO levels with MAKE30 stratified by baseline UPCR

		UPCR < 0.5	UPCR ≥ 0.5	Interaction	*P*-value
		HR (95% CI)	HR (95% CI)	HR (95% CI)	
U-mCHO	Model 1	3.13 (1.40–6.99)	3.09 (2.22–4.29)	1.03 (0.45–2.35)	0.94
	Model 2	3.19 (1.43–7.14)	2.98 (2.12–4.18)	1.04 (0.46–2.38)	0.92
	Model 3	3.06 (1.37–6.85)	2.93 (2.08–4.12)	1.06 (0.46–2.42)	0.89

MAKE, major adverse kidney events; HR, hazard ratio; CI, confidence interval; eGFR, estimated glomerular filtration rate; UPCR, urine protein-to-creatinine ratio; DKD, diabetic kidney disease.

Table 4 shows proteinuria-stratified Cox proportional hazards models (UPCR < 0.5 vs. ≥ 0.5 g/g). This stratification was performed to evaluate effect modification by proteinuria. Within each stratum, Model 1 included age, sex, and baseline eGFR; Model 2 additionally included mean arterial pressure, LDL cholesterol, and DKD status; Model 3 further adjusted for the use of RAS inhibitors, statins, and SGLT2 inhibitors (UPCR was not included as a covariate because the analyses were stratified by UPCR). P for interaction was obtained in the overall cohort by fitting each corresponding model (Models 1–3) including an interaction term between log-transformed U-mCHO and the UPCR stratum indicator. For comparison, the HR for UPCR was derived from a separate overall-cohort Model 3 in which UPCR was modeled as a continuous variable (per 1 g/g): 1.24 (95% CI, 1.12–1.36). Analytic cohort: *n* = 1373.

### Predictive Performance of U-mCHO for Kidney Composite Outcomes

As an exploratory, hypothesis-generating analysis, we evaluated the predictive performance of U-mCHO for MAKE30 and MAKE50 using time-dependent receiver operating characteristic analysis, along with category-free net reclassification improvement and integrated discrimination improvement analyses. These prediction analyses are presented in the Supplementary Materials (Supplementary Figure S5, S6, and S9). Briefly, adding U-mCHO yielded a modest improvement in discrimination, and reclassification metrics were directionally consistent. Findings should be interpreted cautiously because this was a secondary, exploratory analysis without external validation.

### Association Between U-mCHO and Other Covariates

**Figure 4:**
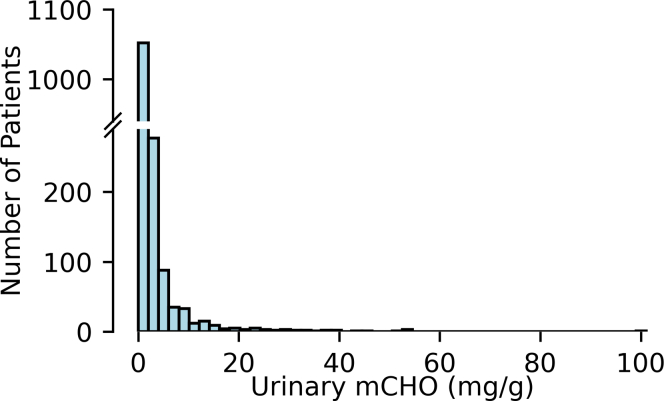
Distribution of U-mCHO levels. Histogram illustrating the right-skewed distribution of urinary microcholesterol (U-mCHO) (mg/g creatinine) among 1562 participants with chronic kidney disease (CKD). The distribution supports subsequent log-transformation for parametric analyses.

To aid interpretation of the prospective findings, we next present cross-sectional associations at baseline, describing correlation structures among U-mCHO and key clinical covariates used for adjustment. The distribution of U-mCHO was right-skewed, and log transformation yielded an approximately normal distribution suitable for parametric analysis (Figure 4). Scatter plots of log-transformed U-mCHO against selected clinical variables, including age, eGFR, UPCR, and LDL cholesterol, are presented in Supplementary Figure S7. In the multivariable linear regression analysis, age, presence of DKD and higher UPCR were independently associated with higher U-mCHO (expressed as percent differences on the original scale), whereas male sex and statin use showed an inverse association (Table 5).

**Table 5:** Multivariable linear regression analysis of factors associated with U-mCHO levels

Variable	Coefficient (% change; 95% CI)	*P-*value
Age (per 10 yrs)	2.30% (1.1–3.5)	< 0.001
Male sex	−14.9% (−17.6 to −12.2)	< 0.001
Mean arterial pressure (mmHg)	0.02% (−0.11 to 0.15)	0.75
UPCR (g/g)	17.2% (16.0–18.5)	< 0.001
eGFR per 10 ml/min per 1.73 m^2^	0.45% (−0.34 to 1.26)	0.27
LDL cholesterol per 10 mg/dl	−0.06% (−0.57 to 0.45)	0.81
RAS inhibitor	−1.61% (−5.0 to 1.9)	0.37
SGLT2 inhibitor	−1.67% (−5.1 to 1.9)	0.35
Statin	−4.64% (−7.7 to −1.5)	0.004
Diabetic kidney disease	7.03% (2.1–12.2)	0.005

Coefficients represent the percent change in U-mCHO per unit increase in each variable, adjusted for all covariates shown. Analytic cohort: *n* = 1373. Abbreviations: eGFR, estimated glomerular filtration rate; RAS, renin–angiotensin system; SGLT2, sodium–glucose cotransporter 2.

## Discussion

In this real-world cohort study of 1562 patients with CKD, higher U-mCHO levels were significantly associated with an earlier onset of the composite kidney outcome, independent of traditional risk factors, including UPCR. Importantly, this association persisted even among patients with low baseline proteinuria (UPCR < 0.5 g/g). In exploratory analyses, time-dependent receiver operating characteristic analysis demonstrated modest improvements in 1-, 2-, and 3-year discrimination when U-mCHO was added to the clinical model.

Dysregulation of lipid metabolism is a well-recognized feature of both CKD and nephrotic syndrome, and its potential contribution to kidney function decline has long been discussed under the concept of “lipid nephrotoxicity”.^17^^,^^18^ Plasma abnormalities in nephrotic syndrome include elevated cholesterol, triglycerides, and apolipoprotein B-containing lipoproteins, along with impaired maturation of HDL particles, reflecting defective reverse cholesterol transport.^19^^,^^20^ Experimental studies have further demonstrated direct lipotoxic injury to mesangial cells,^21^, ^22^, ^23^ podocytes,^24^^,^^25^ and proximal tubular cells,^26^, ^27^, ^28^, ^29^ through mechanisms such as oxidized LDL uptake, fatty acid–induced endoplasmic reticulum stress, and cytoskeletal disruption. Collectively, these observations support the notion that excess lipid exposure may be both a marker and a mediator of kidney injury.

Clinical evidence, however, has been relatively limited. Although circulating lipidomic signatures have been shown to improve CKD risk prediction, urinary lipidomics remains underdeveloped.^30^ Recent prospective studies have linked urinary phosphatidylcholines and sphingomyelins to kidney failure and mortality, but neutral sterols such as cholesterol were not captured due to the methodological limitations of conventional liquid chromatography – mass spectrometry.^2^^,^^9^^,^^10^^,^^13^ Against this backdrop, our study extends previous work by evaluating urinary microcholesterol in a larger, heterogeneous CKD cohort using clinically relevant time-to-event outcomes. Urinary cholesterol, measured using a high-sensitivity enzymatic cycling assay, was independently associated with subsequent kidney outcomes even after adjustment for conventional risk factors, including proteinuria. This builds on previous studies focused on proteinuric glomerular diseases and highlights U-mCHO as a potential addition to the biomarker repertoire.^14^

Importantly, our findings suggest that U-mCHO may provide prognostic information complementary to conventional proteinuria measures. Although UPCR is a well-established prognostic marker in CKD, its apparent association can be attenuated within low-proteinuria strata. In our cohort, U-mCHO remained significantly associated with outcomes (MAKE30) even within the UPCR < 0.5 g/g subgroup. This suggests that U-mCHO may capture aspects of kidney injury that are not fully reflected by proteinuria alone; however, these subgroup findings are exploratory and warrant confirmation in independent cohorts, particularly those with broader representation of earlier-stage or low-proteinuria CKD.

Diabetes and DKD were more prevalent in the highest U-mCHO quartile. This likely reflects, at least in part, the correlation between U-mCHO and proteinuria, because DKD is often accompanied by higher proteinuria. Notably, DKD remained associated with higher U-mCHO even after adjustment for UPCR and other covariates, suggesting that DKD-related factors and/or residual confounding may contribute.

In addition to the prognostic association of U-mCHO, exploratory time-dependent discrimination analyses suggested modest improvement in 1-, 2-, and 3-year performance when U-mCHO was added to the clinical model. These results suggest that U-mCHO may add incremental prognostic information beyond a clinical model based on conventional risk factors^31^; however, they should be interpreted cautiously because the analyses were secondary and no external validation was available. From a clinical standpoint, even small incremental gains in risk stratification may be useful, but the clinical impact and generalizability of these discrimination and reclassification metrics require confirmation in independent cohorts.

From a therapeutic perspective, lipid-lowering interventions, such as statins, have clear cardiovascular benefits in CKD; however, their role in slowing CKD progression remains uncertain.^32^ In our cross-sectional analysis, statin use was associated with lower U-mCHO levels, suggesting a possible pharmacological effect on cholesterol handling. However, this did not translate into improved kidney prognosis, raising the possibility that U-mCHO reflects established kidney injury rather than being a modifiable risk factor. Whether U-mCHO primarily acts as a surrogate for ongoing lipotoxic processes or constitutes a mechanistic mediator warrants further investigation. Future interventional studies targeting lipid metabolism may clarify whether reducing U-mCHO levels can modify the disease trajectory.

This study has several limitations. It was a single-center Japanese cohort and observational in nature; thus, residual confounding, including confounding by indication, cannot be fully excluded. In addition, U-mCHO was measured at a single time point, which limits assessment of within-person temporal variability and time-varying effects; indeed, the association appeared to attenuate over time in sensitivity analyses. Proteinuria was assessed using UPCR rather than urinary albumin-to-creatinine ratio, which may be preferable for risk stratification; in Japan, urinary albumin-to-creatinine ratio testing is not routinely performed in all patients with CKD in part because insurance coverage for measurement is limited in routine practice, and urinary albumin-to-creatinine ratio was therefore not consistently available. Proteinuria adjustment may therefore have been less optimal, potentially leaving residual confounding. Multiple secondary and subgroup analyses were performed, and these should be interpreted as exploratory given the risk of chance findings. Finally, prediction-related analyses were exploratory and lacked external validation, and the underlying biological mechanisms remain to be clarified.

Nonetheless, this study has important strengths. This study represents the largest investigation to date of U-mCHO in a real-world CKD cohort and includes patients with both glomerular and nonglomerular causes of kidney disease, as well as those with low-level proteinuria. This broader inclusion enhances the generalizability of the findings beyond those of previous studies, which were limited to glomerular disease populations.

In summary, U-mCHO showed consistent and independent associations with the composite kidney outcome across a range of clinical settings, including in patients with low proteinuria. It also showed modest incremental improvement in time-dependent discrimination in exploratory analyses, supporting its potential utility as an adjunctive prognostic biomarker alongside conventional measures such as UPCR and warranting further investigation.

## Disclosure

This work was conducted with sponsored research support from Sysmex Corporation provided to the Department of Disease Systems Analysis Medicine, Fujita Health University School of Health Sciences (Principal Investigator: MHoshi). All the other authors declared no competing interests.

## Supplementary Material

Supplementary File (PDF) **Figure S1.** Kaplan–Meier curves for MAKE30. **Figure S2.** Cumulative incidence curves. **Figure S3.** Martingale residual plots. **Figure S4.** Schoenfeld residuals. **Figure S5.** Time-dependent ROC curves. **Figure S6.** Prediction error curves. **Figure S7.** Scatter plots of log(U-mCHO). **Table S1.** Cox models for kidney outcomes (≥ 30% decline in eGFR). **Table S2.** Fine–Gray models and causes of death. **Table S3.** Full Cox proportional hazards model outputs for MAKE50. **Table S4.** Full Cox proportional hazards model outputs for ≥ 50% decline in eGFR. **Table S5.** Full Cox proportional hazards model outputs for initiation of KRT. **Table S6.** Sensitivity models (nonstatin agents). **Table S7.** Landmark and time-varying Cox analyses. **Table S8.** Baseline characteristics (UPCR < 0.5 subgroup). **Table S9.** Prediction metrics (cfNRI/IDI). STROBE checklist.

## Footnotes

Supplementary File (PDF)
**Figure S1.** Kaplan–Meier curves for MAKE30. **Figure S2.** Cumulative incidence curves. **Figure S3.** Martingale residual plots. **Figure S4.** Schoenfeld residuals **Figure S5.** Time-dependent ROC curves. **Figure S6.** Prediction error curves. **Figure S7.** Scatter plots of log(U-mCHO). **Table S1.** Cox models for kidney outcomes (≥ 30% decline in eGFR). **Table S2.** Fine–Gray models and causes of death. **Table S3.** Full Cox proportional hazards model outputs for MAKE50. **Table S4.** Full Cox proportional hazards model outputs for ≥ 50% decline in eGFR. **Table S5.** Full Cox proportional hazards model outputs for initiation of KRT. **Table S6.** Sensitivity models (nonstatin agents). **Table S7.** Landmark and time-varying Cox analyses. **Table S8.** Baseline characteristics (UPCR < 0.5 subgroup). **Table S9.** Prediction metrics (cfNRI/IDI). STROBE checklist.
