# Supplementary material for: Urinary Microcholesterol and Adverse Kidney Outcomes in CKD
Source: Kidney Int Rep. 2026 Apr 21;11(8):106549. doi: 10.1016/j.ekir.2026.106549 (PMC13273664; doi:10.1016/j.ekir.2026.106549)
Supplement: Supplementary File (PDF) — Figure S1. Kaplan–Meier curves for MAKE30. Figure S2. Cumulative incidence curves. Figure S3. Martingale residual plots. Figure S4. Schoenfeld residuals. Figure S5. Time-dependent ROC curves. Figure S6. Prediction error curves. Figure S7. Scatter plots of log(U-mCHO). Table S1. Cox models for kidney outcomes (≥ 30% decline in eGFR). Table S2. Fine–Gray models and causes of death. Table S3. Full Cox proportional hazards model outputs for MAKE50. Table S4. Full Cox proportional hazards model outputs for ≥ 50% decline in eGFR. Table S5. Full Cox proportional hazards model outputs for initiation of KRT. Table S6. Sensitivity models (nonstatin agents). Table S7. Landmark and time-varying Cox analyses. Table S8. Baseline characteristics (UPCR < 0.5 subgroup). Table S9. Prediction metrics (cfNRI/IDI). STROBE checklist. [file mmc1.pdf]

Supplemental materials.

Table of Contents

Supplementary Figure S1: Kaplan–Meier curves for MAKE30 according to U-mCHO quartiles p3

Supplementary Figure S2: Cumulative incidence function curves of MAKE50 by U-mCHO quartiles p4

Supplementary Figure S3: Martingale residual plots to assess linearity of continuous covariates p5

Supplementary Figure S4: Schoenfeld residuals to assess the proportional hazards assumption p6

Supplementary Figure S5: Time-dependent ROC curves for MAKE30 and MAKE50 (base model vs model including U-mCHO).. p7

Supplementary Figure S6: Prediction error curves for models with and without U-mCHO......................... p8

Supplementary Figure S7: Association between U-mCHO and clinical variables p9

Supplementary Table S1. Hazard ratios for kidney outcomes (≥30% decline in eGFR) according to U-mCHO p11

Supplementary Table S2. Fine–Gray subdistribution hazard ratios for MAKE50 by U-mCHO quartiles (non–kidney-related death as a competing event) p12

Supplementary Table S3. Full Cox proportional hazards model outputs for MAKE50 by U-mCHO specification (quartiles, trend, and continuous log-transformed) p.14

Supplementary Table S4. Full Cox proportional hazards model outputs for ≥50% decline in eGFR by U-mCHO specification (quartiles, trend, and continuous log-transformed).. p.17

Supplementary Table S5. Full Cox proportional hazards model outputs for initiation of KRT by U-mCHO specification (quartiles, trend, and continuous log-transformed) p.20

Supplementary Table S6. Association between U-mCHO quartiles and MAKE50 after additional adjustment for lipid-lowering agents (statin and non-statin)... p23

Supplementary Table S7. Sensitivity analyses for potential non-proportional hazards in the association between U-mCHO and MAKE50: landmark Cox analyses at 365 and 730 days and a time-varying coefficient model... p24

Supplementary Table S8. Baseline characteristics of the study population, overall and stratified by baseline proteinuria (UPCR <0.5 g/g vs. ≥0.5 g/g) p.27

Supplementary Table S9: Incremental predictive performance of adding U-mCHO to the clinical model (Model 3) for 1-, 2-, 3-year prediction of MAKE50 .... p.30

STROBE checklist p.31

Supplementary Figure S1: Kaplan–Meier curves for MAKE30 according to U-mCHO quartiles

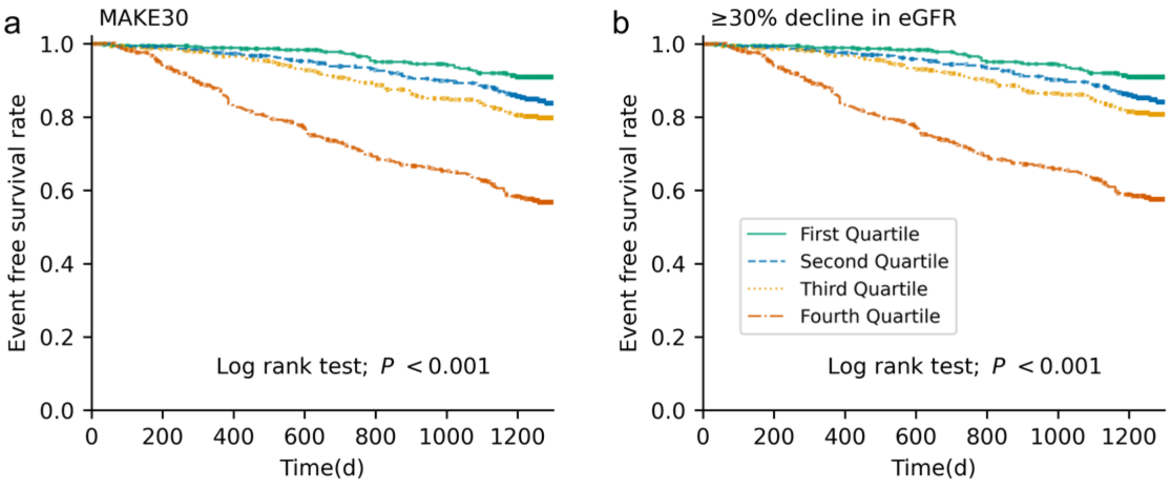

(a) At risk

At risk	0	200	400	600	800	1000	1200
Q1	391	371	358	341	317	304	265
Q2	390	367	349	328	309	291	245
Q3	390	365	349	326	301	272	240
Q4	391	343	287	259	219	196	160

(b) At risk

At risk	0	200	400	600	800	1000	1200
Q1	391	371	358	341	318	305	265
Q2	390	369	352	331	313	294	248
Q3	390	365	350	327	302	273	240
Q4	391	343	287	259	219	196	160

Legend:

Kaplan–Meier curves showing cumulative event-free survival for kidney outcomes according to urinary micro-cholesterol (U-mCHO) quartiles. (a) Composite kidney outcome (MAKE30), defined as a ≥30% decline in estimated glomerular filtration rate (eGFR), initiation of kidney replacement therapy (KRT), or kidney-related death. (b) ≥30% decline in eGFR. The highest U-mCHO quartile exhibited the greatest risk of adverse kidney outcomes (log-rank $p < 0.001$ for both panels). Numbers at risk are shown below each panel under the corresponding Kaplan–Meier curve. Analytic cohort: n = 1,562.

Abbreviations: MAKE, major adverse kidney events; MAKE30, composite of a ≥30% decline in eGFR, initiation of kidney replacement therapy, and kidney-related death; U-mCHO, urinary micro-cholesterol.

Supplementary Figure S2: Cumulative incidence function curves of MAKE50 by U-mCHO quartiles

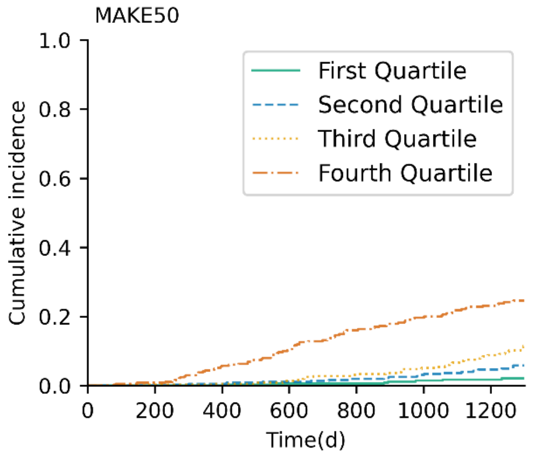

At risk

Q1	391	373	362	344	331	316	284
Q2	390	369	355	338	324	312	273
Q3	390	369	358	343	324	299	266
Q4	391	361	326	301	263	238	211

Legend:

Cumulative incidence function (CIF) curves for major adverse kidney events defined by a ≥50% decline in eGFR (MAKE50) according to quartiles of baseline urinary micro-cholesterol (U-mCHO). Non–kidney-related death was treated as a competing event. Numbers at risk are shown below the plot. Analytic cohort: n = 1,562.

Abbreviations: MAKE, major adverse kidney events; MAKE50, composite of a ≥50% decline in eGFR, initiation of kidney replacement therapy, and kidney-related death; U-mCHO, urinary micro-cholesterol.

Supplementary Figure S3: Martingale residual plots to assess linearity of continuous covariates

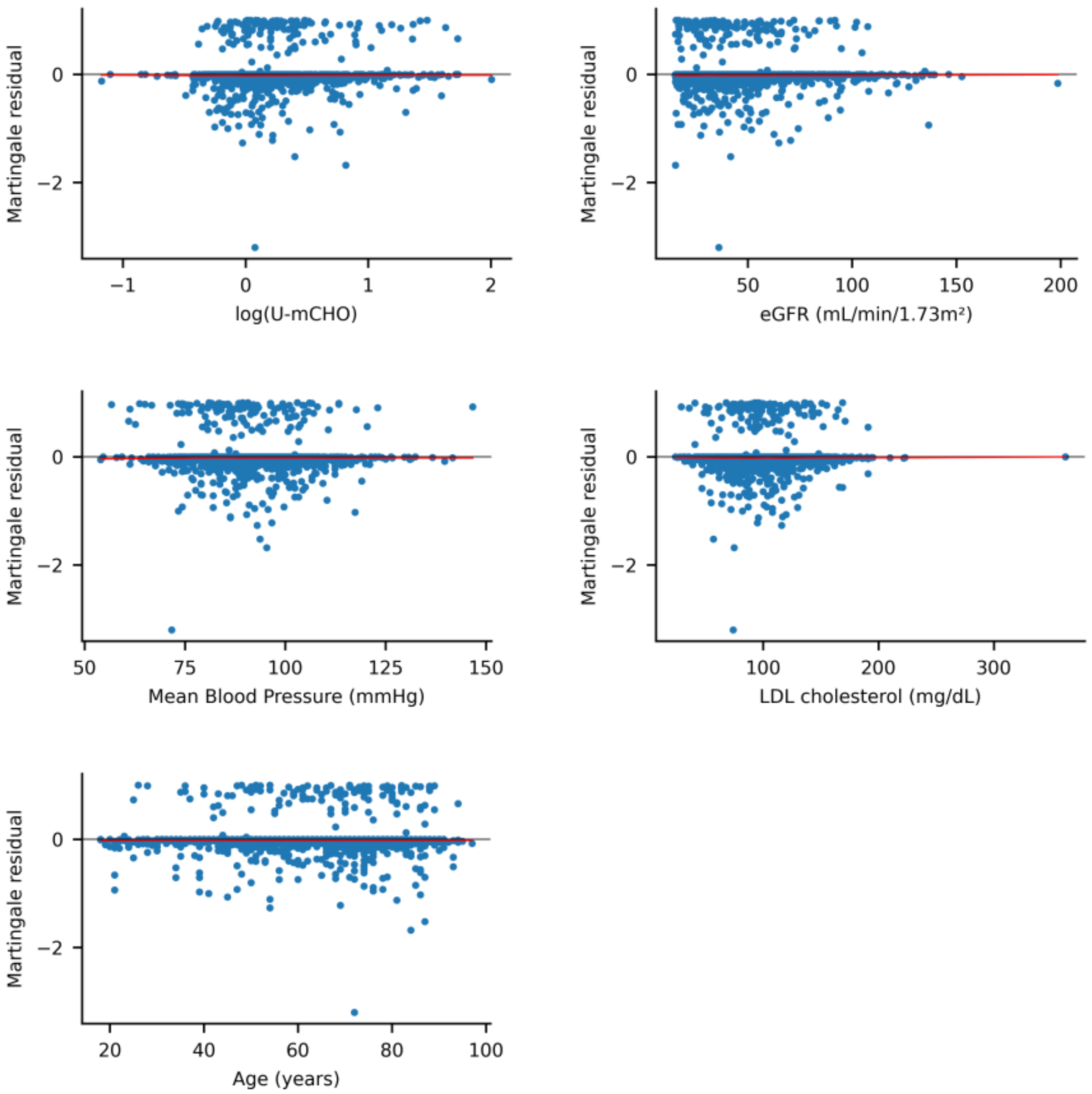

Legend:

Martingale residuals from the Cox proportional hazards model (Model 3) plotted against continuous predictors to assess the linearity assumption. Panels show log10-transformed urinary micro-cholesterol (log(U-mCHO)), baseline eGFR, mean blood pressure, LDL cholesterol, and age. The red line indicates a LOWESS-smoothed trend. Based on these diagnostics, continuous variables were modeled as linear terms in the primary Cox models.

Abbreviations: eGFR, estimated glomerular filtration rate; LDL, low-density lipoprotein; U-mCHO, urinary micro-cholesterol; LOWESS, locally weighted scatterplot smoothing.

Supplementary Figure S4: Schoenfeld residuals to assess the proportional hazards assumption

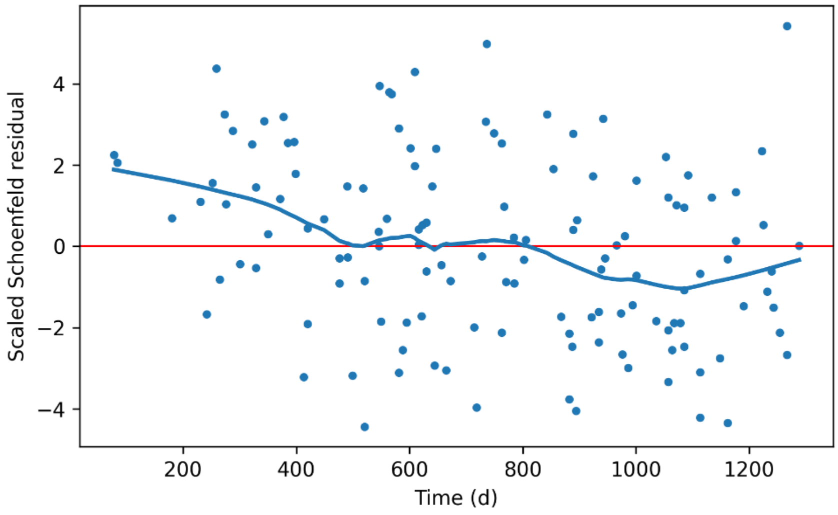

Legend:

Scaled Schoenfeld residuals for log10-transformed urinary micro-cholesterol (U-mCHO) plotted against follow-up time to evaluate the proportional hazards assumption in the Cox model (Model 3). The solid line shows a smoothed trend over time; deviation from a horizontal pattern suggests potential time-varying effects. Analytic cohort: n = 1,373.

Supplementary Figure S5: Time-dependent ROC curves for MAKE30 and MAKE50 (base model vs model including U-mCHO)

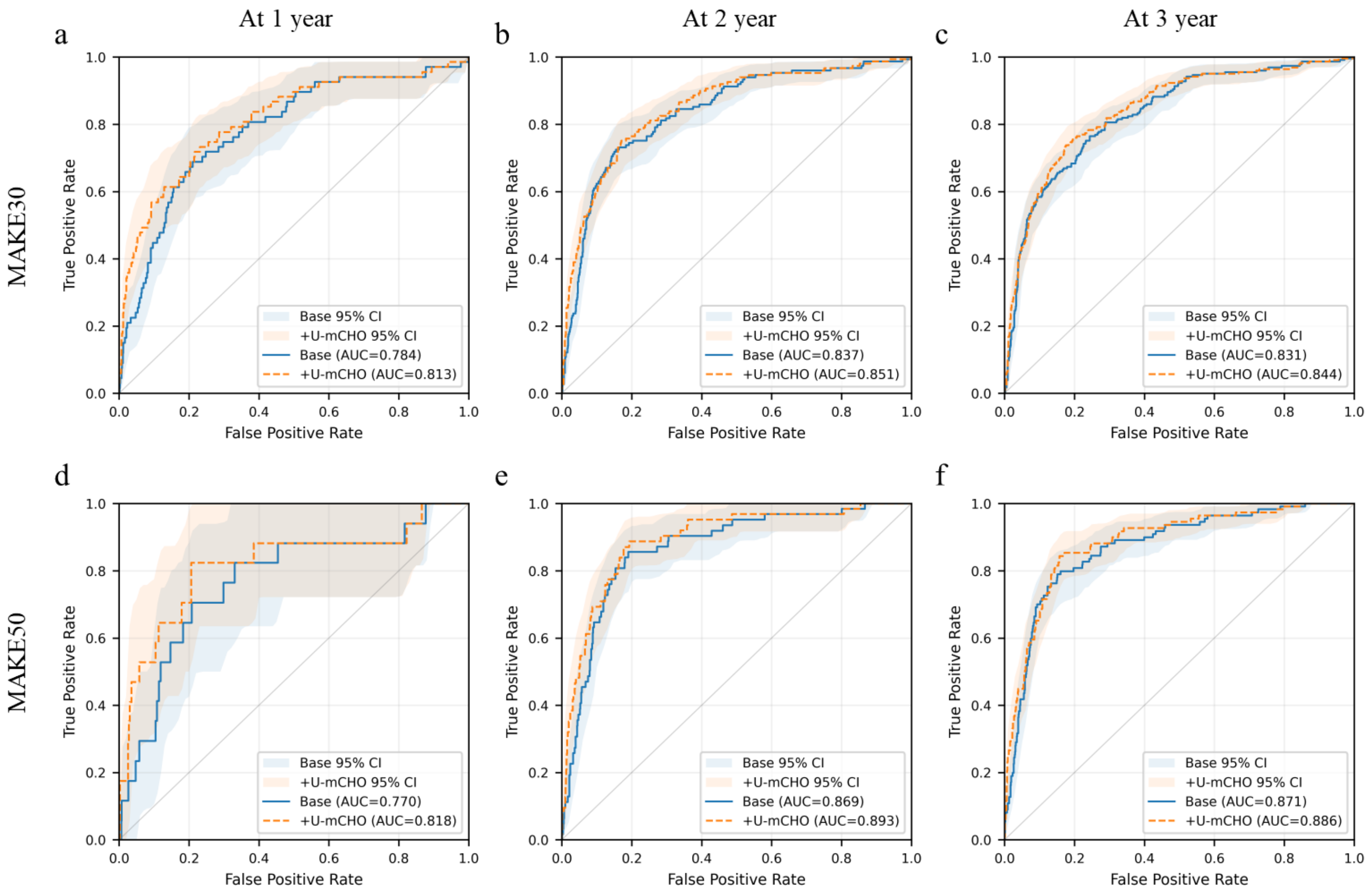

Legend:

Time-dependent receiver operating characteristic (ROC) curves are shown for MAKE30 (a–c) and MAKE50 (d–f) at 1 year (a, d), 2 years (b, e), and 3 years (c, f). The solid blue lines represent the base model (Model 3 without U-mCHO), and the dashed orange lines represent Model 3 including U-mCHO. ROC curves were constructed using out-of-fold predictions from 10-fold cross-validation. Shaded areas indicate 95% confidence intervals estimated from 2,000 bootstrap resamples. Analytic cohort: n = 1,373. Abbreviations: MAKE, major adverse kidney events; MAKE30/MAKE50, composite of a ≥30%/≥50% decline in eGFR, initiation of kidney replacement therapy, or kidney-related death; AUC, area under the curve; CI, confidence interval; U-mCHO, urinary micro-cholesterol.

Supplementary Figure S6: Prediction error curves for models with and without U-mCHO

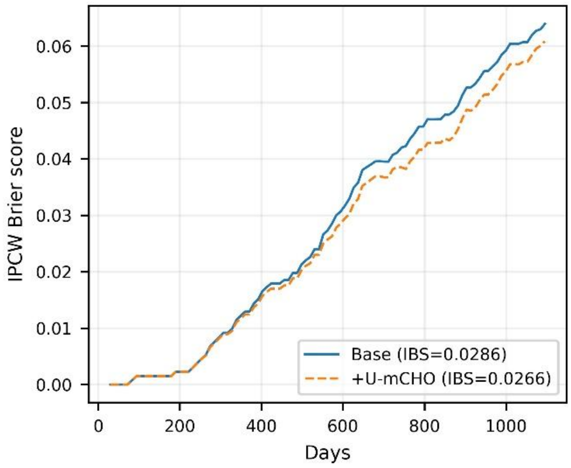

Legend:

Time-dependent prediction error curves based on the inverse probability of censoring weighted (IPCW) Brier score comparing the base clinical model (solid blue line) and the model additionally including urinary micro-cholesterol (U-mCHO) (dashed orange line). Lower values indicate better prediction accuracy. Integrated Brier score (IBS): base model 0.0286; +U-mCHO model 0.0266 (ΔIBS = 0.0020). Analytic cohort: n = 1,373.

Abbreviations: IPCW, inverse probability of censoring weighting; IBS, integrated Brier score; U-mCHO, urinary micro-cholesterol.

Supplementary Figure S7: Association between U-mCHO and clinical variables

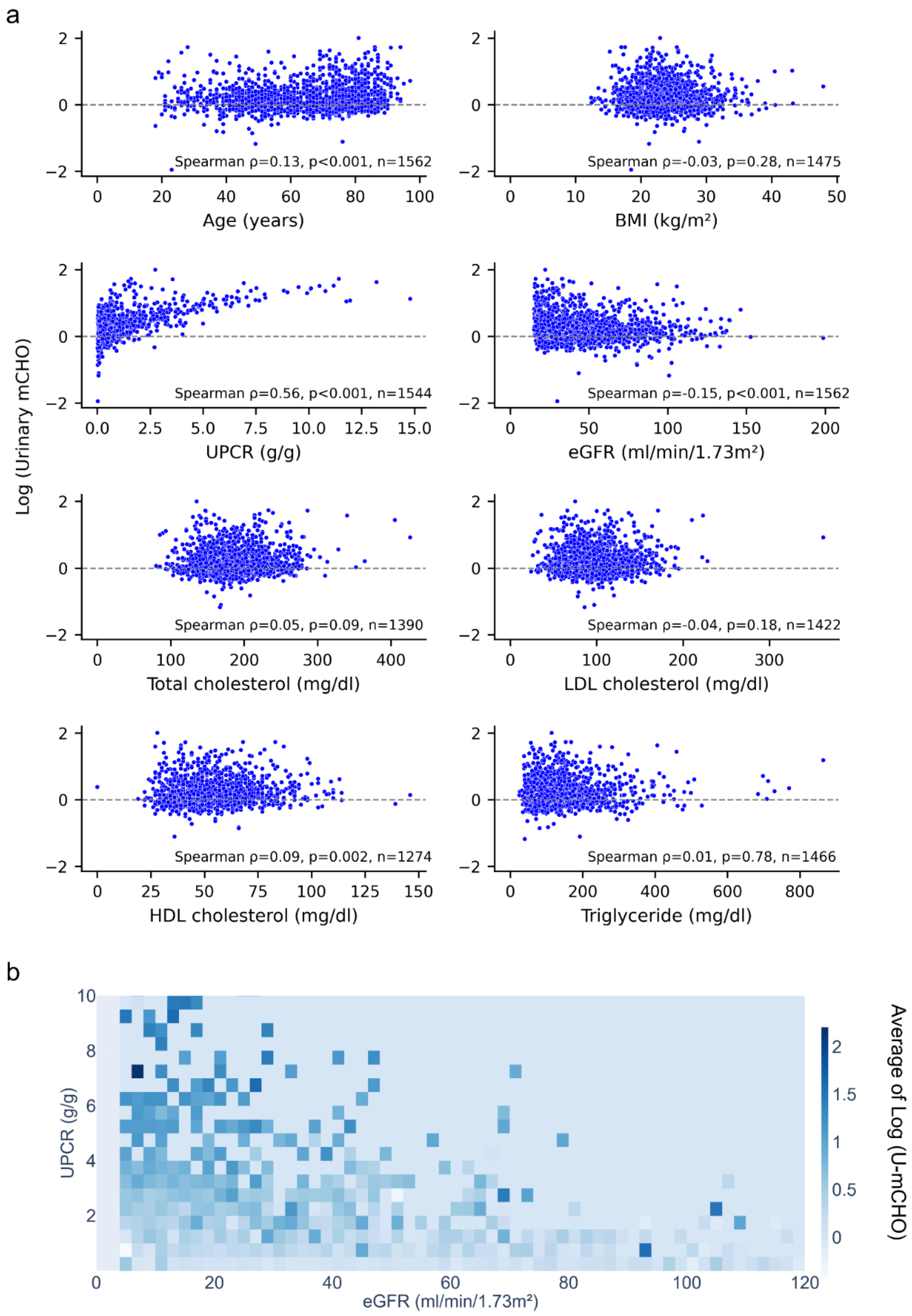

Legend:

(A) Scatter plots of log10-transformed urinary micro-cholesterol (U-mCHO) levels against age, body mass index (BMI), urinary protein-to-creatinine ratio (UPCR), estimated glomerular filtration rate (eGFR), total cholesterol, low-density lipoprotein cholesterol, high-density lipoprotein cholesterol, and triglyceride levels. Each dot represents an individual patient; dashed lines indicate reference at zero. Spearman's rank correlation coefficients (ρ) and p values are shown in each panel; n indicates the number of observations.

(B) Density heatmap of log10-transformed U-mCHO levels averaged across strata of eGFR and UPCR.

Supplementary Table S1. Hazard ratios for kidney outcomes (≥30% decline in eGFR) according to U-mCHO

Outcome	Q1 U-mCHO	Q2 U-mCHO	Q3 U-mCHO	Q4 U-mCHO	P for trend (HR per 1-quartile increase)		Continuous	
	HR (95% CI)	HR (95% CI)	HR (95% CI)	HR (95% CI)	HR (95% CI)	*P* value	HR (95% CI)	*P* value
≥30% decline in eGFR								
Model 1	1.0 (ref)	1.80 (1.10–2.96)	1.93 (1.17–3.19)	3.68 (2.26–6.00)	1.51 (1.31–1.74)	<0.001	3.04 (2.24–4.14)	<0.001
Model 2	1.0 (ref)	1.84 (1.12–3.01)	1.96 (1.19–3.24)	3.64 (2.22–5.95)	1.50 (1.29–1.73)	<0.001	2.98 (2.18–4.09)	<0.001
Model 3	1.0 (ref)	1.81 (1.11–2.97)	1.95 (1.19–3.22)	3.65 (2.24–5.96)	1.50 (1.30–1.73)	<0.001	2.94 (2.14–4.03)	<0.001

Footnote:

HRs and 95% CIs were derived from Cox proportional hazards models. Model 1 included age, sex, baseline eGFR, and UPCR; Model 2 additionally included mean arterial pressure, LDL cholesterol, and diabetic nephropathy status; and Model 3 further adjusted for the use of RAS inhibitors, statins, and SGLT2 inhibitors. Q1 U-mCHO served as the reference category. P for trend was assessed by modeling U-mCHO quartiles as an ordinal variable (1–4); the reported HR represents the change in hazard per 1-quartile increase. U-mCHO was also analyzed as a log10-transformed continuous variable. MAKE30 was defined as a composite of a ≥30% decline in eGFR, initiation of kidney replacement therapy, or kidney-related death. Continuous HR is expressed per 1-unit increase in log10-transformed U-mCHO (log(U-mCHO)).

Analytic cohort: n = 1,373.

Abbreviations: MAKE, major adverse kidney events; KRT, kidney replacement therapy; eGFR, estimated glomerular filtration rate; HR, hazard ratio; CI, confidence interval; NA, not applicable; U-mCHO, urinary micro-cholesterol.

Supplementary Table S2. Fine–Gray subdistribution hazard ratios for MAKE50 by U-mCHO quartiles (non–kidney-related death as a competing event)

Panel A. Fine–Gray subdistribution hazard ratios for MAKE50

Outcome	Q1 U-mCHO		Q2 U-mCHO		Q3 U-mCHO		Q4 U-mCHO		P for trend (HR per 1-quartile increase)		Continuous	
	sHR (95% CI)	*P* value	sHR (95% CI)	*P* value	sHR (95% CI)	*P* value	sHR (95% CI)	*P* value	sHR (95% CI)	*P* value	sHR (95% CI)	*P* value
**Primary endpoint (MAKE50)**												
Model 1	1.0 (ref)	–	2.57 (1.03–6.42)	0.044	3.38 (1.35–8.47)	0.009	6.55 (2.69–15.95)	<0.001	1.75 (1.42–2.17)	<0.001	3.52 (2.27–5.45)	<0.001
Model 2	1.0 (ref)	–	2.59 (1.03–6.50)	0.043	3.46 (1.37–8.74)	0.009	6.53 (2.65–16.06)	<0.001	1.74 (1.40–2.16)	<0.001	3.41 (2.18–5.35)	<0.001
Model 3	1.0 (ref)	–	2.53 (1.01–6.38)	0.049	3.48 (1.38–8.78)	0.008	6.50 (2.64–16.02)	<0.001	1.74 (1.41–2.16)	<0.001	3.37 (2.14–5.33)	<0.001

Panel B. Number of non–kidney-related deaths, n

Non–kidney-related deaths, n	
Overall	99
Q1 U-mCHO	17
Q2 U-mCHO	25
Q3 U-mCHO	27
Q4 U-mCHO	30

Footnote;

Panel A shows sHRs and 95% CIs derived from Fine–Gray subdistribution hazards models treating non–kidney-related death as a competing event. Model 1 included age, sex, baseline eGFR, and UPCR; Model 2 additionally included mean arterial pressure, LDL cholesterol, and diabetic nephropathy status; and Model 3 further

adjusted for the use of RAS inhibitors, statins, and SGLT2 inhibitors. Q1 U-mCHO served as the reference category. P for trend was assessed by modeling U-mCHO quartiles as an ordinal variable (1–4); the reported HR represents the change in hazard per 1-quartile increase. U-mCHO was also analyzed as a log10-transformed continuous variable. MAKE50 was defined as a composite of a ≥50% decline in eGFR, initiation of kidney replacement therapy, or kidney-related death. Continuous sHR is expressed per 1-unit increase in log10-transformed U-mCHO (log(U-mCHO)). Panel B shows the number of non–kidney-related deaths overall and by U-mCHO quartile in the analytic cohort. Analytic cohort: n = 1,373.

Abbreviations: MAKE, major adverse kidney events; KRT, kidney replacement therapy; eGFR, estimated glomerular filtration rate; sHR, subdistribution hazard ratio; CI, confidence interval; UPCR, urine protein-to-creatinine ratio; RAS, renin–angiotensin system; SGLT2, sodium–glucose cotransporter 2; U-mCHO, urinary micro-cholesterol.

Supplementary Table S3. Full Cox proportional hazards model outputs for MAKE50 by U-mCHO specification (quartiles, trend, and continuous log-transformed).

Panel A: Quartile-based U-mCHO model

	Model 1			Model 2			Model 3		
	β	HR (95% CI)	*P* value	β	HR (95% CI)	*P* value	β	HR (95% CI)	*P* value
Age (per 10 years)	-0.093	0.91 (0.80–1.04)	0.155	-0.100	0.90 (0.79–1.03)	0.136	-0.094	0.91 (0.80–1.04)	0.172
Sex: Male (ref = Female)	0.746	2.11 (1.43–3.10)	<0.001	0.677	1.97 (1.33–2.91)	<0.001	0.654	1.92 (1.30–2.85)	0.001
UPCR category (g/g): 0.15–0.5 (ref = <0.15)	0.238	1.27 (0.51–3.13)	0.606	0.214	1.24 (0.50–3.06)	0.644	0.214	1.24 (0.50–3.07)	0.644
UPCR category (g/g): ≥0.5 (ref = <0.15)	1.446	4.25 (1.94–9.31)	<0.001	1.372	3.94 (1.79–8.71)	<0.001	1.374	3.95 (1.78–8.79)	<0.001
eGFR (per 10 ml/min/1.73m^2)	-0.562	0.57 (0.49–0.66)	<0.001	-0.534	0.59 (0.50–0.68)	<0.001	-0.517	0.60 (0.51–0.69)	<0.001
Mean arterial pressure (mmHg)	–	–	–	0.014	1.01 (1.00–1.03)	0.111	0.012	1.01 (0.99–1.03)	0.171
LDL Cholesterol (per 10 mg/dl)	–	–	–	-0.063	0.94 (0.89–1.00)	0.035	-0.064	0.94 (0.88–1.00)	0.037
Diabetic kidney disease (DKD): Yes (ref = No)	–	–	–	0.191	1.21 (0.81–1.81)	0.349	0.225	1.25 (0.83–1.88)	0.279
RAS inhibitor use: Yes (ref = No)	–	–	–	–	–	–	0.380	1.46 (0.85–2.52)	0.172
Statin use: Yes (ref = No)	–	–	–	–	–	–	-0.082	0.92 (0.63–1.35)	0.675
SGLT2 inhibitor use: Yes (ref = No)	–	–	–	–	–	–	-0.264	0.77 (0.52–1.13)	0.185
Q2 U-mCHO (ref = Q1)	1.064	2.90 (1.06–7.94)	0.039	1.067	2.91 (1.06–7.98)	0.038	1.030	2.80 (1.02–7.70)	0.046
Q3 U-mCHO (ref = Q1)	1.277	3.59 (1.32–9.72)	0.012	1.294	3.65 (1.34–9.90)	0.011	1.279	3.59 (1.32–9.75)	0.012
Q4 U-mCHO (ref = Q1)	2.010	7.47 (2.81–19.83)	<0.001	2.002	7.41 (2.78–19.75)	<0.001	1.988	7.30 (2.74–19.49)	<0.001

Model fit statistics	Model 1	Model 2	Model 3
AIC	1535.6	1534.3	1536.6
C-index	0.873	0.875	0.876

Panel B: Trend model with U-mCHO quartiles entered as an ordinal variable (per +1 quartile)

	Model 1			Model 2			Model 3		
	β	HR (95% CI)	*P* value	β	HR (95% CI)	*P* value	β	HR (95% CI)	*P* value
Age (per 10 years)	-0.094	0.91 (0.80–1.03)	0.149	-0.102	0.90 (0.79–1.03)	0.129	-0.097	0.91 (0.79–1.04)	0.155
Sex: Male (ref = Female)	0.738	2.09 (1.42–3.08)	<0.001	0.667	1.95 (1.32–2.88)	<0.001	0.646	1.91 (1.29–2.82)	0.001
UPCR category (g/g): 0.15–0.5 (ref = <0.15)	0.264	1.30 (0.53–3.21)	0.567	0.241	1.27 (0.52–3.14)	0.602	0.240	1.27 (0.51–3.14)	0.603
UPCR category (g/g): ≥0.5 (ref = <0.15)	1.451	4.27 (1.95–9.33)	<0.001	1.385	3.99 (1.81–8.79)	<0.001	1.388	4.01 (1.81–8.88)	<0.001
eGFR (per 10 ml/min/1.73m^2)	-0.564	0.57 (0.49–0.66)	<0.001	-0.534	0.59 (0.50–0.68)	<0.001	-0.518	0.60 (0.51–0.69)	<0.001
Mean arterial pressure (mmHg)	–	–	–	0.013	1.01 (1.00–1.03)	0.123	0.011	1.01 (0.99–1.03)	0.188
LDL Cholesterol (per 10 mg/dl)	–	–	–	-0.064	0.94 (0.88–0.99)	0.032	-0.065	0.94 (0.88–1.00)	0.035
Diabetic kidney disease (DKD): Yes (ref = No)	–	–	–	0.196	1.22 (0.82–1.81)	0.336	0.229	1.26 (0.84–1.88)	0.267
RAS inhibitor use: Yes (ref = No)	–	–	–	–	–	–	0.379	1.46 (0.85–2.51)	0.172
Statin use: Yes (ref = No)	–	–	–	–	–	–	-0.078	0.93 (0.63–1.36)	0.692
SGLT2 inhibitor use: Yes (ref = No)	–	–	–	–	–	–	-0.279	0.76 (0.51–1.12)	0.160
U-mCHO quartile (per +1 quartile increase)	0.588	1.80 (1.44–2.26)	<0.001	0.580	1.79 (1.42–2.25)	<0.001	0.583	1.79 (1.42–2.25)	<0.001

**Model fit statistics**	**Model 1**	**Model 2**	**Model 3**
AIC	1533.4	1532.0	1534.0
C-index	0.873	0.875	0.876

Panel C: Continuous model with log-transformed U-mCHO (per 1-unit increase in log U-mCHO)

	Model 1			Model 2			Model 3		
	β	HR (95% CI)	*P* value	β	HR (95% CI)	*P* value	β	HR (95% CI)	*P* value
Age (per 10 years)	-0.121	0.89 (0.78–1.01)	0.068	-0.123	0.88 (0.77–1.01)	0.069	-0.117	0.89 (0.78–1.02)	0.093
Sex: Male (ref = Female)	0.702	2.02 (1.38–2.96)	<0.001	0.612	1.84 (1.25–2.71)	0.002	0.590	1.80 (1.22–2.66)	0.003
UPCR category (g/g): 0.15–0.5 (ref = <0.15)	0.309	1.36 (0.55–3.35)	0.500	0.283	1.33 (0.54–3.27)	0.538	0.276	1.32 (0.53–3.25)	0.549
UPCR category (g/g): ≥0.5 (ref = <0.15)	1.514	4.54 (2.10–9.83)	<0.001	1.456	4.29 (1.97–9.32)	<0.001	1.460	4.31 (1.96–9.46)	<0.001
eGFR (per 10 ml/min/1.73m^2)	-0.569	0.57 (0.49–0.66)	<0.001	-0.545	0.58 (0.50–0.67)	<0.001	-0.534	0.59 (0.50–0.68)	<0.001
Mean arterial pressure (mmHg)	–	–	–	0.013	1.01 (1.00–1.03)	0.121	0.012	1.01 (1.00–1.03)	0.157
LDL Cholesterol (per 10 mg/dl)	–	–	–	-0.058	0.94 (0.89–1.00)	0.042	-0.058	0.94 (0.89–1.00)	0.046
Diabetic kidney disease (DKD): Yes (ref = No)	–	–	–	0.146	1.16 (0.78–1.71)	0.467	0.169	1.18 (0.79–1.76)	0.405
RAS inhibitor use: Yes (ref = No)	–	–	–	–	–	–	0.296	1.34 (0.78–2.31)	0.286
Statin use: Yes (ref = No)	–	–	–	–	–	–	-0.025	0.98 (0.67–1.42)	0.896
SGLT2 inhibitor use: Yes (ref = No)	–	–	–	–	–	–	-0.168	0.85 (0.57–1.25)	0.403
Log U-mCHO (continuous)	1.378	3.97 (2.64–5.97)	<0.001	1.358	3.89 (2.57–5.90)	<0.001	1.313	3.72 (2.45–5.65)	<0.001

Model fit statistics	Model 1	Model 2	Model 3
AIC	1523.1	1522.7	1526.9
C-index	0.877	0.878	0.879

Footnote:

HRs, 95% CIs, and β coefficients were derived from Cox proportional hazards models. Outcome was MAKE50, defined as a composite of a ≥50% decline in eGFR, initiation of kidney replacement therapy, or kidney-related death. Model 1 included age, sex, baseline eGFR, and UPCR; Model 2 additionally included mean arterial pressure, LDL cholesterol, and DKD status; and Model 3 further adjusted for the use of RAS inhibitors, statins, and SGLT2 inhibitors. In the quartile-based analyses, Q1 U-mCHO served as the reference category. In the trend analyses, U-mCHO quartiles were entered as an ordinal variable (per +1 quartile increase). In the continuous analyses, U-mCHO was entered as a log10-transformed continuous variable, and the HR is expressed per 1-unit increase in log10-transformed U-mCHO. Model fit statistics (AIC and C-index) are provided under each panel. Analytic cohort: n = 1,373. Abbreviations: MAKE, major adverse kidney events; KRT, kidney replacement therapy; eGFR, estimated glomerular filtration rate; UPCR, urine protein-to-creatinine ratio; HR, hazard ratio; CI, confidence interval; DKD, diabetic kidney disease; RAS, renin–angiotensin system; SGLT2, sodium–glucose cotransporter 2; AIC, Akaike information criterion; NA, not applicable.

Supplementary Table S4. Full Cox proportional hazards model outputs for ≥50% decline in eGFR by U-mCHO specification (quartiles, trend, and continuous log-transformed).

Panel A: Quartile-based U-mCHO model

	Model 1			Model 2			Model 3		
	**β**	**HR (95% CI)**	***P* value**	**β**	**HR (95% CI)**	***P* value**	**β**	**HR (95% CI)**	***P* value**
Age (per 10 years)	-0.114	0.89 (0.78–1.02)	0.096	-0.122	0.88 (0.77–1.02)	0.083	-0.120	0.89 (0.77–1.02)	0.096
Sex: Male (ref = Female)	0.621	1.86 (1.24–2.78)	0.002	0.535	1.71 (1.14–2.56)	0.010	0.520	1.68 (1.12–2.53)	0.012
UPCR category (g/g): 0.15–0.5 (ref = <0.15)	1.226	3.41 (0.94–12.31)	0.062	1.191	3.29 (0.91–11.90)	0.070	1.191	3.29 (0.91–11.92)	0.070
UPCR category (g/g): ≥0.5 (ref = <0.15)	2.560	12.93 (3.89–42.97)	<0.001	2.461	11.72 (3.51–39.14)	<0.001	2.476	11.89 (3.53–40.04)	<0.001
eGFR (per 10 ml/min/1.73m^2)	-0.501	0.61 (0.52–0.70)	<0.001	-0.467	0.63 (0.54–0.73)	<0.001	-0.445	0.64 (0.55–0.75)	<0.001
Mean arterial pressure (mmHg)	–	–	–	0.020	1.02 (1.00–1.04)	0.028	0.018	1.02 (1.00–1.04)	0.052
LDL Cholesterol (per 10 mg/dl)	–	–	–	-0.063	0.94 (0.88–1.00)	0.046	-0.059	0.94 (0.89–1.00)	0.066
Diabetic kidney disease (DKD): Yes (ref = No)	–	–	–	0.362	1.44 (0.94–2.18)	0.090	0.374	1.45 (0.95–2.23)	0.086
RAS inhibitor use: Yes (ref = No)	–	–	–	–	–	–	0.319	1.38 (0.77–2.47)	0.283
Statin use: Yes (ref = No)	–	–	–	–	–	–	0.056	1.06 (0.70–1.60)	0.794
SGLT2 inhibitor use: Yes (ref = No)	–	–	–	–	–	–	-0.332	0.72 (0.47–1.08)	0.114
Q2 U-mCHO (ref = Q1)	0.746	2.11 (0.76–5.84)	0.152	0.759	2.13 (0.77–5.93)	0.146	0.716	2.04 (0.73–5.70)	0.171
Q3 U-mCHO (ref = Q1)	0.805	2.24 (0.82–6.14)	0.117	0.825	2.28 (0.83–6.27)	0.110	0.813	2.26 (0.82–6.21)	0.115
Q4 U-mCHO (ref = Q1)	1.614	5.02 (1.90–13.30)	0.001	1.586	4.88 (1.83–13.02)	0.002	1.560	4.76 (1.78–12.72)	0.002

**Model fit statistics**	**Model 1**	**Model 2**	**Model 3**
AIC	1362.8	1358.5	1360.9
C-index	0.879	0.882	0.884

Panel B: Trend model with U-mCHO quartiles entered as an ordinal variable (per +1 quartile)

	Model 1			Model 2			Model 3		
	β	HR (95% CI)	*P* value	β	HR (95% CI)	*P* value	β	HR (95% CI)	*P* value
Age (per 10 years)	-0.112	0.89 (0.78–1.02)	0.101	-0.121	0.89 (0.77–1.02)	0.086	-0.123	0.88 (0.77–1.02)	0.089
Sex: Male (ref = Female)	0.612	1.84 (1.23–2.76)	0.003	0.524	1.69 (1.12–2.54)	0.012	0.512	1.67 (1.11–2.51)	0.014
UPCR category (g/g): 0.15–0.5 (ref = <0.15)	1.228	3.41 (0.95–12.31)	0.060	1.198	3.31 (0.92–11.96)	0.067	1.193	3.30 (0.91–11.91)	0.069
UPCR category (g/g): ≥0.5 (ref = <0.15)	2.520	12.43 (3.77–41.01)	<0.001	2.433	11.40 (3.44–37.79)	<0.001	2.451	11.60 (3.47–38.77)	<0.001
eGFR (per 10 ml/min/1.73m^2)	-0.503	0.60 (0.52–0.70)	<0.001	-0.467	0.63 (0.54–0.73)	<0.001	-0.447	0.64 (0.55–0.74)	<0.001
Mean arterial pressure (mmHg)	–	–	–	0.020	1.02 (1.00–1.04)	0.031	0.017	1.02 (1.00–1.04)	0.057
LDL Cholesterol (per 10 mg/dl)	–	–	–	-0.064	0.94 (0.88–1.00)	0.044	-0.059	0.94 (0.89–1.00)	0.066
Diabetic kidney disease (DKD): Yes (ref = No)	–	–	–	0.382	1.47 (0.97–2.22)	0.073	0.393	1.48 (0.97–2.26)	0.069
RAS inhibitor use: Yes (ref = No)	–	–	–	–	–	–	0.312	1.37 (0.76–2.44)	0.293
Statin use: Yes (ref = No)	–	–	–	–	–	–	0.067	1.07 (0.71–1.62)	0.754
SGLT2 inhibitor use: Yes (ref = No)	–	–	–	–	–	–	-0.348	0.71 (0.47–1.07)	0.097
U-mCHO quartile (per +1 quartile increase)	0.527	1.69 (1.33–2.15)	<0.001	0.504	1.66 (1.30–2.11)	<0.001	0.502	1.65 (1.30–2.10)	<0.001

Model fit statistics	Model 1	Model 2	Model 3
AIC	1361.2	1356.5	1358.6
C-index	0.877	0.880	0.883

Panel C: Continuous model with log-transformed U-mCHO (per 1-unit increase in log U-mCHO)

	Model 1			Model 2			Model 3		
	β	HR (95% CI)	*P* value	β	HR (95% CI)	*P* value	β	HR (95% CI)	*P* value
Age (per 10 years)	-0.142	0.87 (0.76–0.99)	0.042	-0.144	0.87 (0.75–1.00)	0.043	-0.144	0.87 (0.75–1.00)	0.049
Sex: Male (ref = Female)	0.600	1.82 (1.22–2.72)	0.003	0.501	1.65 (1.10–2.47)	0.015	0.488	1.63 (1.09–2.44)	0.018
UPCR category (g/g): 0.15–0.5 (ref = <0.15)	1.250	3.49 (0.97–12.56)	0.056	1.217	3.38 (0.94–12.16)	0.063	1.209	3.35 (0.93–12.09)	0.065
UPCR category (g/g): ≥0.5 (ref = <0.15)	2.519	12.41 (3.80–40.58)	<0.001	2.436	11.42 (3.48–37.50)	<0.001	2.454	11.63 (3.51–38.53)	<0.001
eGFR (per 10 ml/min/1.73m^2)	-0.509	0.60 (0.52–0.70)	<0.001	-0.478	0.62 (0.53–0.72)	<0.001	-0.462	0.63 (0.54–0.73)	<0.001
Mean arterial pressure (mmHg)	–	–	–	0.019	1.02 (1.00–1.04)	0.031	0.018	1.02 (1.00–1.04)	0.045
LDL Cholesterol (per 10 mg/dl)	–	–	–	-0.061	0.94 (0.89–1.00)	0.045	-0.056	0.95 (0.89–1.00)	0.064
Diabetic kidney disease (DKD): Yes (ref = No)	–	–	–	0.316	1.37 (0.91–2.07)	0.133	0.327	1.39 (0.91–2.11)	0.125
RAS inhibitor use: Yes (ref = No)	–	–	–	–	–	–	0.241	1.27 (0.71–2.27)	0.415
Statin use: Yes (ref = No)	–	–	–	–	–	–	0.113	1.12 (0.74–1.69)	0.587
SGLT2 inhibitor use: Yes (ref = No)	–	–	–	–	–	–	-0.246	0.78 (0.52–1.18)	0.246
Log U-mCHO (continuous)	1.352	3.51 (2.26–5.45)	<0.001	1.313	3.69 (2.38–5.73)	<0.001	1.261	3.84 (2.50–5.91)	<0.001

**Model fit statistics**	**Model 1**	**Model 2**	**Model 3**
AIC	1347.8	1344.4	1348.2
C-index	0.882	0.884	0.886

Footnote:

HRs, 95% CIs, and β coefficients were derived from Cox proportional hazards models. Outcome was ≥50% decline in eGFR. Model 1 included age, sex, baseline eGFR, and UPCR; Model 2 additionally included mean arterial pressure, LDL cholesterol, and DKD status; and Model 3 further adjusted for the use of RAS inhibitors, statins, and SGLT2 inhibitors. In the quartile-based analyses, Q1 U-mCHO served as the reference category. In the trend analyses, U-mCHO quartiles were entered as an ordinal variable (per +1 quartile increase). In the continuous analyses, U-mCHO was entered as a log10-transformed continuous variable, and the HR is expressed per 1-unit increase in log10-transformed U-mCHO. Model fit statistics (AIC and C-index) are provided under each panel. Analytic cohort: n = 1,373. Abbreviations: MAKE, major adverse kidney events; KRT, kidney replacement therapy; eGFR, estimated glomerular filtration rate; UPCR, urine protein-to-creatinine ratio; HR, hazard ratio; CI, confidence interval; DKD, diabetic kidney disease; RAS, renin–angiotensin system; SGLT2, sodium–glucose cotransporter 2; AIC, Akaike information criterion; NA, not applicable.

Supplementary Table S5. Full Cox proportional hazards model outputs for initiation of KRT by U-mCHO specification (quartiles, trend, and continuous log-transformed).

Panel A: Quartile-based U-mCHO model

	Model 1			Model 2			Model 3		
	β	HR (95% CI)	*P* value	β	HR (95% CI)	*P* value	β	HR (95% CI)	*P* value
Age (per 10 years)	-0.307	0.74 (0.62–0.87)	<0.001	-0.316	0.73 (0.61–0.87)	<0.001	-0.301	0.74 (0.62–0.88)	<0.001
Sex: Male (ref = Female)	1.095	2.99 (1.72–5.21)	<0.001	0.994	2.70 (1.54–4.73)	<0.001	0.985	2.68 (1.52–4.70)	<0.001
UPCR category (g/g): 0.15–0.5 (ref = <0.15)	-1.968	0.14 (0.02–1.21)	0.074	-2.097	0.12 (0.01–1.07)	0.058	-2.133	0.12 (0.01–1.03)	0.054
UPCR category (g/g): ≥0.5 (ref = <0.15)	0.843	2.32 (0.81–6.66)	0.117	0.628	1.87 (0.63–5.57)	0.258	0.574	1.77 (0.59–5.32)	0.306
eGFR (per 10 ml/min/1.73m^2)	-1.019	0.36 (0.27–0.48)	<0.001	-0.972	0.38 (0.28–0.50)	<0.001	-0.967	0.38 (0.28–0.51)	<0.001
Mean arterial pressure (mmHg)	–	–	–	0.026	1.03 (1.00–1.05)	0.030	0.025	1.02 (1.00–1.05)	0.037
LDL Cholesterol (per 10 mg/dl)	–	–	–	-0.087	0.92 (0.84–0.99)	0.035	-0.086	0.92 (0.84–1.00)	0.044
Diabetic kidney disease (DKD): Yes (ref = No)	–	–	–	0.277	1.32 (0.79–2.20)	0.290	0.248	1.28 (0.76–2.17)	0.358
RAS inhibitor use: Yes (ref = No)	–	–	–	–	–	–	0.383	1.47 (0.65–3.29)	0.352
Statin use: Yes (ref = No)	–	–	–	–	–	–	0.076	1.08 (0.62–1.88)	0.788
SGLT2 inhibitor use: Yes (ref = No)	–	–	–	–	–	–	-0.053	0.95 (0.56–1.62)	0.847
Q2 U-mCHO (ref = Q1)	0.307	1.36 (0.32–5.70)	0.675	0.339	1.40 (0.33–5.92)	0.645	0.322	1.38 (0.32–5.87)	0.662
Q3 U-mCHO (ref = Q1)	0.365	1.44 (0.35–5.99)	0.616	0.409	1.51 (0.36–6.30)	0.576	0.409	1.51 (0.36–6.33)	0.577
Q4 U-mCHO (ref = Q1)	1.978	7.23 (1.94–26.89)	0.003	1.991	7.32 (1.94–27.65)	0.003	1.965	7.13 (1.88–27.11)	0.004

**Model fit statistics**	**Model 1**	**Model 2**	**Model 3**
AIC	757.7	754.6	759.5
C-index	0.931	0.935	0.935

Panel B: Trend model with U-mCHO quartiles entered as an ordinal variable (per +1 quartile)

	Model 1			Model 2			Model 3		
	β	HR (95% CI)	*P* value	β	HR (95% CI)	*P* value	β	HR (95% CI)	*P* value
Age (per 10 years)	-0.279	0.76 (0.64–0.89)	<0.001	-0.293	0.75 (0.63–0.89)	<0.001	-0.282	0.75 (0.63–0.90)	0.002
Sex: Male (ref = Female)	1.081	2.95 (1.69–5.14)	<0.001	0.969	2.63 (1.50–4.61)	<0.001	0.962	2.62 (1.49–4.60)	<0.001
UPCR category (g/g): 0.15–0.5 (ref = <0.15)	-2.042	0.13 (0.02–1.12)	0.063	-2.162	0.12 (0.01–1.00)	0.050	-2.184	0.11 (0.01–0.98)	0.048
UPCR category (g/g): ≥0.5 (ref = <0.15)	0.608	1.84 (0.67–5.00)	0.234	0.401	1.49 (0.53–4.22)	0.449	0.365	1.44 (0.50–4.11)	0.495
eGFR (per 10 ml/min/1.73m^2)	-0.996	0.37 (0.28–0.49)	<0.001	-0.949	0.39 (0.29–0.51)	<0.001	-0.937	0.39 (0.30–0.52)	<0.001
Mean arterial pressure (mmHg)	–	–	–	0.025	1.03 (1.00–1.05)	0.034	0.024	1.02 (1.00–1.05)	0.045
LDL Cholesterol (per 10 mg/dl)	–	–	–	-0.089	0.92 (0.84–0.99)	0.034	-0.085	0.92 (0.84–1.00)	0.049
Diabetic kidney disease (DKD): Yes (ref = No)	–	–	–	0.362	1.44 (0.86–2.40)	0.166	0.328	1.39 (0.82–2.35)	0.221
RAS inhibitor use: Yes (ref = No)	–	–	–	–	–	–	0.393	1.48 (0.66–3.31)	0.338
Statin use: Yes (ref = No)	–	–	–	–	–	–	0.103	1.11 (0.64–1.93)	0.717
SGLT2 inhibitor use: Yes (ref = No)	–	–	–	–	–	–	-0.128	0.88 (0.52–1.49)	0.631
U-mCHO quartile (per +1 quartile increase)	0.925	2.52 (1.75–3.64)	<0.001	0.906	2.47 (1.70–3.59)	<0.001	0.893	2.44 (1.68–3.54)	<0.001

Model fit statistics	Model 1	Model 2	Model 3
AIC	759.9	756.2	760.8
C-index	0.929	0.931	0.933

Panel C: Continuous model with log-transformed U-mCHO (per 1-unit increase in log U-mCHO)

	Model 1			Model 2			Model 3		
	β	HR (95% CI)	*P* value	β	HR (95% CI)	*P* value	β	HR (95% CI)	*P* value
Age (per 10 years)	-0.322	0.72 (0.61–0.86)	<0.001	-0.324	0.72 (0.61–0.86)	<0.001	-0.317	0.73 (0.61–0.87)	<0.001
Sex: Male (ref = Female)	1.027	2.79 (1.61–4.86)	<0.001	0.896	2.45 (1.40–4.28)	0.002	0.903	2.47 (1.41–4.33)	0.002
UPCR category (g/g): 0.15–0.5 (ref = <0.15)	-1.943	0.14 (0.02–1.23)	0.077	-2.023	0.13 (0.02–1.14)	0.066	-2.090	0.12 (0.01–1.07)	0.058
UPCR category (g/g): ≥0.5 (ref = <0.15)	0.779	2.18 (0.81–5.88)	0.124	0.625	1.87 (0.68–5.13)	0.226	0.544	1.72 (0.61–4.83)	0.301
eGFR (per 10 ml/min/1.73m^2)	-1.020	0.36 (0.27–0.48)	<0.001	-0.974	0.38 (0.29–0.50)	<0.001	-0.980	0.38 (0.28–0.50)	<0.001
Mean arterial pressure (mmHg)	–	–	–	0.025	1.02 (1.00–1.05)	0.033	0.025	1.03 (1.00–1.05)	0.032
LDL Cholesterol (per 10 mg/dl)	–	–	–	-0.077	0.93 (0.86–1.00)	0.053	-0.073	0.93 (0.86–1.01)	0.069
Diabetic kidney disease (DKD): Yes (ref = No)	–	–	–	0.294	1.34 (0.81–2.21)	0.251	0.251	1.29 (0.77–2.13)	0.333
RAS inhibitor use: Yes (ref = No)	–	–	–	–	–	–	0.263	1.30 (0.58–2.91)	0.523
Statin use: Yes (ref = No)	–	–	–	–	–	–	0.215	1.24 (0.73–2.12)	0.432
SGLT2 inhibitor use: Yes (ref = No)	–	–	–	–	–	–	0.016	1.02 (0.60–1.73)	0.952
Log U-mCHO (continuous)	1.830	6.24 (3.62–10.74)	<0.001	1.785	5.96 (3.41–10.39)	<0.001	1.772	5.88 (3.33–10.40)	<0.001

Model fit statistics	Model 1	Model 2	Model 3
AIC	750.7	748.6	753.4
C-index	0.933	0.936	0.937

Footnote:

HRs, 95% CIs, and β coefficients were derived from Cox proportional hazards models. Outcome was initiation of KRT. Model 1 included age, sex, baseline eGFR, and UPCR; Model 2 additionally included mean arterial pressure, LDL cholesterol, and DKD status; and Model 3 further adjusted for the use of RAS inhibitors, statins, and SGLT2 inhibitors. In the quartile-based analyses, Q1 U-mCHO served as the reference category. In the trend analyses, U-mCHO quartiles were entered as an ordinal variable (per +1 quartile increase). In the continuous analyses, U-mCHO was entered as a log10-transformed continuous variable, and the HR is expressed per 1-unit increase in log10-transformed U-mCHO. Model fit statistics (AIC and C-index) are provided under each panel. Analytic cohort: n = 1,373. Abbreviations: MAKE, major adverse kidney events; KRT, kidney replacement therapy; eGFR, estimated glomerular filtration rate; UPCR, urine protein-to-creatinine ratio; HR, hazard ratio; CI, confidence interval; DKD, diabetic kidney disease; RAS, renin–angiotensin system; SGLT2, sodium–glucose cotransporter 2; AIC, Akaike information criterion; NA, not applicable.

Supplementary Table S6. Association between U-mCHO quartiles and MAKE50 after additional adjustment for lipid-lowering agents (statin and non-statin)

Outcome	HR (95% CI)	*P* value
**Primary endpoint (MAKE50)**		
U-mCHO 1st quartile	1.0 (ref)	–
U-mCHO 2nd quartile	2.64 (1.03–6.73)	0.043
U-mCHO 3rd quartile	3.55 (1.41–8.95)	0.007
U-mCHO 4th quartile	6.90 (2.78–17.17)	<0.001
Use of statin	0.99 (0.66–1.48)	0.955
Use of lipid-lowering agent without statin	0.91 (0.42–1.97)	0.807

Footnote:

HRs and 95% CIs were derived from Cox proportional hazards models. This model corresponds to Model 3 with additional adjustment for lipid-lowering therapy: baseline use of statins and baseline use of lipid-lowering agents without statins (non-statin agents grouped as a single category). Model 3 covariates were age, sex, mean arterial pressure, baseline eGFR, UPCR, LDL cholesterol, DKD status, and baseline use of RAS inhibitors, SGLT2 inhibitors, and statin. Q1 U-mCHO served as the reference category. Analytic cohort: n = 1,373.

Abbreviations: U-mCHO, urinary micro-cholesterol; MAKE50, major adverse kidney events defined by a ≥50% decline in eGFR, initiation of kidney replacement therapy, or kidney-related death; eGFR, estimated glomerular filtration rate; UPCR, urine protein-to-creatinine ratio; LDL, low-density lipoprotein; DKD, diabetic kidney disease; RAS, renin–angiotensin system; SGLT2, sodium–glucose cotransporter 2; HR, hazard ratio; CI, confidence interval.

Supplementary Table S7. Sensitivity analyses for potential non-proportional hazards in the association between U-mCHO and MAKE50: landmark Cox analyses at 365 and 730 days and a time-varying coefficient model

Panel A. Landmark Cox proportional hazards models.

Outcome	Q1 U-mCHO		Q2 U-mCHO		Q3 U-mCHO		Q4 U-mCHO		P for trend (HR per 1-quartile increase)		Continuous	
	HR (95% CI)	*P* value	HR (95% CI)	*P* value	HR (95% CI)	*P* value	HR (95% CI)	*P* value	HR (95% CI)	*P* value	HR (95% CI)	*P* value
**Primary endpoint (MAKE50)**												
**After 365 days**												
Model 1	1.0 (ref)	–	2.21 (0.90–5.39)	0.082	3.59 (1.51–8.53)	0.004	5.67 (2.40–13.36)	<0.001	1.75 (1.42–2.17)	<0.001	3.55 (2.35–5.38)	<0.001
Model 2	1.0 (ref)	–	2.13 (0.87–5.25)	0.100	3.12 (1.30–7.51)	0.011	5.29 (2.22–12.61)	<0.001	1.74 (1.40–2.16)	<0.001	3.41 (2.22–5.23)	<0.001
Model 3	1.0 (ref)	–	2.03 (0.82–5.01)	0.125	3.08 (1.28–7.40)	0.012	5.12 (2.14–12.22)	<0.001	1.74 (1.41–2.16)	<0.001	3.22 (2.09–4.95)	<0.001
**After 730 days**												
Model 1	1.0 (ref)	–	2.58 (0.91–7.32)	0.074	4.55 (1.64–12.57)	0.004	5.43 (1.94–15.23)	0.001	1.58 (1.22–2.05)	0.001	3.12 (1.80–5.40)	<0.001
Model 2	1.0 (ref)	–	2.42 (0.84–6.93)	0.101	3.77 (1.34–10.60)	0.012	4.81 (1.69–13.71)	0.003	1.54 (1.18–2.03)	0.002	2.94 (1.66–5.21)	<0.001
Model 3	1.0 (ref)	–	2.27 (0.79–6.53)	0.128	3.75 (1.34–10.55)	0.012	4.58 (1.60–13.10)	0.005	1.53 (1.16–2.01)	0.002	2.69 (1.53–4.75)	<0.001

Panel B. Summarizes the numbers at risk and events at each landmark

Landmark	Quartile	n	MAKE50	Non-kidney-related death
365	Q1 U-mCHO	364	7	15
	Q2 U-mCHO	357	18	14
	Q3 U-mCHO	360	35	17
	Q4 U-mCHO	331	69	18
730	Q1 U-mCHO	335	5	9
	Q2 U-mCHO	328	14	4
	Q3 U-mCHO	328	26	10
	Q4 U-mCHO	274	35	10

Panel C. Time-varying coefficient model for urinary mCHO (log(U-mCHO) × log(time/365))

Model	Time interaction coefficient: log(U-mCHO) × log(time/365)	Wald P	LRT P
Model 1	-0.95	0.01	0.01
Model 2	-0.80	0.03	0.03
Model 3	-0.81	0.03	0.02

Footnote:

Panel A shows HRs (95% CIs) from landmark Cox proportional hazards models. For the 365-day landmark, analyses included participants who were alive and event-free at day 365 and were followed from day 365 onward; for the 730-day landmark, analyses included participants who were alive and event-free at day 730 and were followed from day 730 onward. Model 1 included age, sex, baseline eGFR, and UPCR; Model 2 additionally included mean arterial pressure, LDL cholesterol, and diabetic nephropathy status; and Model 3 further adjusted for the use of RAS inhibitors, statins, and SGLT2 inhibitors. Q1 U-mCHO served as the reference category. P for trend was assessed by modeling U-mCHO quartiles as an ordinal variable (1–4); the reported HR represents the change in hazard per 1-quartile increase. U-mCHO was also

analyzed as a log10-transformed continuous variable. Continuous HR is expressed per 1-unit increase in log10-transformed U-mCHO (log(U-mCHO)). MAKE50 was defined as a composite of a ≥50% decline in eGFR, initiation of kidney replacement therapy, or kidney-related death. Panel B summarizes the numbers at risk and events at each landmark (by U-mCHO quartile). Panel C summarizes a time-varying coefficient Cox model including an interaction between log10(U-mCHO) and log(time/365), where time is measured in days. Wald P tests the interaction term, and LRT P compares models with versus without the interaction term. Negative interaction coefficients indicate attenuation of the association over follow-up.

Abbreviations: U-mCHO, urinary micro-cholesterol; MAKE50, major adverse kidney events defined by a ≥50% decline in eGFR, initiation of kidney replacement therapy, or kidney-related death; eGFR, estimated glomerular filtration rate; UPCR, urine protein-to-creatinine ratio; LDL, low-density lipoprotein; RAS, renin–angiotensin system; SGLT2, sodium–glucose cotransporter 2; HR, hazard ratio; CI, confidence interval; LRT, likelihood ratio test.

Supplementary Table S8. Baseline characteristics of the study population, overall and stratified by baseline proteinuria (UPCR <0.5 g/g vs. ≥0.5 g/g)

Variable	Subcategory	Overall	UPCR <0.5 g/g	UPCR ≥0.5 g/g
Number		1562	995	567
Age		64 (17)	63 (17)	67 (17)
Sex, female		704 (45%)	469 (47%)	235 (41%)
BMI		23.6 (4.2)	23.3 (4.0)	24.1 (4.4)
Smoking	current	151 (10%)	87 (9%)	64 (11%)
	never	788 (50%)	523 (53%)	265 (47%)
	past	495 (32%)	304 (31%)	191 (34%)
Mean arterial pressure, mmHg		91.9 (12.3)	90.8 (11.9)	93.8 (12.8)
Albumin, g/dl		3.9 (0.4)	4.0 (0.3)	3.7 (0.4)
BUN, mg/dl		24.1 (12.1)	22.2 (10.8)	27.6 (13.4)
eGFR, ml/min/1.73m^2		47 (24)	51 (24)	41 (23)
CKD stage	G1	100 (6%)	78 (8%)	22 (4%)
	G2	283 (18%)	197 (20%)	86 (15%)
	G3a	340 (22%)	254 (26%)	86 (15%)
	G3b	415 (27%)	282 (28%)	133 (23%)
	G4	424 (27%)	184 (18%)	240 (42%)
Total cholesterol, mg/dl		186 (39)	186 (38)	186 (42)
LDL cholesterol, mg/dl		99 (31)	99 (30)	99 (33)
HDL cholesterol, mg/dl		54 (17)	55 (18)	53 (16)
Triglyceride, mg/dl		143 (88)	135 (78)	155 (100)
UPCR, g/g		0.9 (1.6)	0.2 (0.1)	2.1 (2.1)
U-mCHO, mg/g		3.0 (5.8)	1.5 (1.3)	5.6 (8.8)

Supplementary Table S8 (continued)

Variable	Subcategory	Overall	UPCR <0.5 g/g	UPCR ≥0.5 g/g
Cause of kidney disease	Diabetic kidney disease	232 (15%)	96 (10%)	136 (24%)
	Nephrosclerosis	254 (16%)	180 (18%)	74 (13%)
	Glomerulonephritis	600 (38%)	399 (40%)	201 (35%)
	Others	476 (30%)	320 (32%)	156 (28%)
Diabetes mellitus		432 (28%)	216 (22%)	216 (38%)
Drugs				
Antihypertensive agents		1307 (84%)	775 (78%)	532 (94%)
RAS inhibitor		1062 (68%)	604 (61%)	458 (81%)
ARNI		163 (10%)	88 (9%)	75 (13%)
SGLT2 inhibitor		454 (29%)	229 (23%)	225 (40%)
GLP-1 agonist		65 (4%)	23 (2%)	42 (7%)
Lipid-lowering agents		939 (60%)	559 (56%)	380 (67%)
Statin		792 (51%)	460 (46%)	332 (59%)
Fibrate		18 (1%)	9 (0.9%)	9 (2%)
PPARα modulator		49 (3%)	26 (3%)	23 (4%)
Cholesterol absorption inhibitor		153 (10%)	85 (9%)	68 (12%)
PCSK9 inhibitor		7 (0.4%)	3 (0.3%)	4 (0.7%)
Omega-3 fatty acids		209 (13%)	130 (13%)	79 (14%)
Probucol		2 (0.1%)	2 (0.2%)	0 (0.0%)
Nicotinic acid		36 (2%)	27 (3%)	9 (2%)
Resin		7 (0.4%)	3 (0.3%)	4 (0.7%)

Footnote;

Values are presented as mean (SD) or n (%). CKD stage was defined by baseline eGFR (ml/min/1.73 m^2): G1 ≥90; G2 60–89; G3a 45–59; G3b 30–44; G4 15–29; G5 <15. Abbreviations: BMI, body mass index; eGFR, estimated glomerular filtration rate; CKD, chronic kidney disease; BUN, blood urea nitrogen; UPCR, urine protein-to-creatinine ratio; U-mCHO, urinary micro-cholesterol; RAS, renin–angiotensin system; ARNI, angiotensin receptor–neprilysin inhibitor; SGLT2, sodium–glucose cotransporter 2; GLP-1, glucagon-like peptide-1; PPAR, peroxisome proliferator–activated receptor; PCSK9, proprotein convertase subtilisin/kexin type 9.

Supplementary Table S9: Incremental predictive performance of adding U-mCHO to the clinical model (Model 3) for 1-, 2-, 3-year prediction of MAKE50

Time (months)	ΔAUC	95% CI	P value	cfNRI	95% CI	P value	IDI	95% CI	P value
12	0.048	0.029–0.068	<0.001	1.06	0.70–1.33	<0.001	0.04	0.01–0.06	<0.001
24	0.024	0.011–0.037	<0.001	0.64	0.40–0.87	<0.001	0.06	0.04–0.09	<0.001
36	0.015	0.006–0.025	0.003	0.38	0.20–0.57	<0.001	0.05	0.03–0.08	<0.001

Footnote;

Values are estimates with 95% confidence intervals. Category-free net reclassification improvement (cfNRI) and integrated discrimination improvement (IDI) were calculated using 2,000 bootstrap resamples. Analytic cohort: n = 1,373.

Abbreviations: MAKE, major adverse kidney events; MAKE50, composite of a ≥50% decline in eGFR, initiation of kidney replacement therapy, or kidney-related death; AUC, area under the curve; CI, confidence interval; cfNRI, category-free net reclassification improvement; IDI, integrated discrimination improvement; U-mCHO, urinary micro-cholesterol.

STROBE Statement—Checklist of items that should be included in reports of ***cohort studies***

	Item No	Recommendation	Comment
**Title and abstract**	1	(*a*) Indicate the study's design with a commonly used term in the title or the abstract	Described in the title as "a cohort study," fulfilling the design identification requirement.
		(*b*) Provide in the abstract an informative and balanced summary of what was done and what was found	The abstract summarizes background, methods, results, and conclusions with key numerical findings.
**Introduction**			
Background/rationale	2	Explain the scientific background and rationale for the investigation being reported	The *Introduction* explains the background linking urinary lipids and kidney injury, providing the rationale for investigating urinary mCHO.
Objectives	3	State specific objectives, including any prespecified hypotheses	The last paragraph of the *Introduction* clearly states the study objectives: to examine the association between urinary mCHO and kidney outcomes.
**Methods**			
Study design	4	Present key elements of study design early in the paper	The *Methods* section begins by stating that this is a cohort study of CKD outpatients with prospective measurement of urinary mCHO and follow-up for outcomes.
Setting	5	Describe the setting, locations, and relevant dates, including periods of recruitment, exposure, follow-up, and data collection	Described in the *Methods (Patients)* section: single-center study at Fujita Health University Hospital; mCHO measured April–July 2022; follow-up through December 2025 via electronic records.
Participants	6	(*a*) Give the eligibility criteria, and the sources and methods of selection of participants. Describe methods of follow-up	Eligibility criteria and exclusion process described in *Methods (Patients)* and shown in Figure 1; follow-up procedures described in the same section.
		(*b*) For matched studies, give matching criteria and number of exposed and unexposed	Not applicable.
Variables	7	Clearly define all outcomes, exposures, predictors, potential confounders, and effect modifiers. Give diagnostic criteria, if applicable	All study variables, including outcomes (MAKE50 and components), exposure (urinary mCHO), and covariates (e.g., age, sex, eGFR, UPCR, LDL-C, DKD status, medications), are defined in the

			Methods (Outcome, Statistical Analysis) sections.
Data sources/ measurement	8*	For each variable of interest, give sources of data and details of methods of assessment (measurement). Describe comparability of assessment methods if there is more than one group	Measurement methods for urinary mCHO (enzymatic cycling, analyzer model, wavelengths) and data sources for clinical variables are detailed in the *Methods (Measurement of urinary micro-cholesterol, Clinical evaluation)* sections.
Bias	9	Describe any efforts to address potential sources of bias	Potential bias was addressed through multivariable adjustment, stratified analyses, and restriction (eGFR ≥ 15 ml/min/1.73 m²); these procedures are described in the *Statistical Analysis* section, and limitations are discussed in the *Discussion*.
Study size	10	Explain how the study size was arrived at	The *Methods (Patients)* section explains that all eligible CKD outpatients with available mCHO measurements during April–July 2022 were included, yielding 1,562 participants.
Quantitative variables	11	Explain how quantitative variables were handled in the analyses. If applicable, describe which groupings were chosen and why	Handling of quantitative variables is described in the *Statistical Analysis* section: urinary mCHO analyzed as quartiles and log-transformed continuous; UPCR used continuously and categorically; rationale for these groupings stated.
Statistical methods	12	(*a*) Describe all statistical methods, including those used to control for confounding	The *Statistical Analysis* section details Cox proportional hazards models with sequential adjustment (Models 1–3) for potential confounders, and time-dependent ROC with IPCW and bootstrapping.
		(*b*) Describe any methods used to examine subgroups and interactions	Subgroup and interaction analyses are described in the *Statistical Analysis* section, examining age, sex, BMI, eGFR, LDL-C, DKD, and medication use.
		(*c*) Explain how missing data were addressed	Missing data handling is described in the *Statistical Analysis* section: complete-case analysis was applied, with missingness is about 12%.
		(*d*) If applicable, explain how loss to follow-up was addressed	Follow-up procedures and censoring (at outcome, death, or transfer) are described in the *Outcome* section.
		(*e*) Describe any sensitivity analyses	Sensitivity analyses are described in the *Results (Predictive performance of*

			urinary mCHO) and *Supplementary Materials*, where analyses for MAKE30, CIF and Fine Gray analyses were done to confirm robustness of the main findings.
**Results**			
Participants	13*	(a) Report numbers of individuals at each stage of study—eg numbers potentially eligible, examined for eligibility, confirmed eligible, included in the study, completing follow-up, and analysed	Numbers at each stage (screening, exclusions, final n = 1,562) are described in the *Result (Baseline Clinical Characteristics of the Study Population)* section and illustrated in Figure 1.
		(b) Give reasons for non-participation at each stage	Reasons for exclusion (non-CKD, advanced CKD, KRT, AKI, missing data, age < 18) are listed in the *Result (Baseline Clinical Characteristics of the Study Population)* section.
		(c) Consider use of a flow diagram	A participant flow diagram is provided as Figure 1.
Descriptive data	14*	(a) Give characteristics of study participants (eg demographic, clinical, social) and information on exposures and potential confounders	Baseline characteristics, exposures, and potential confounders are presented in Table 1 and described in the *Results (Baseline Clinical Characteristics)* section.
		(b) Indicate number of participants with missing data for each variable of interest	Numbers with missing data for each variable are mentioned in the *Methods (Statistical Analysis)* section (12%).
		(c) Summarise follow-up time (eg, average and total amount)	Follow-up duration (median 41.6 months) and range (IQR 33.6-42.8) are reported in the *Results (Urinary mCHO and kidney prognosis)* section.
Outcome data	15*	Report numbers of outcome events or summary measures over time	Numbers of MAKE30/50 events and components are shown in Table 2 and described in the *Results (Urinary mCHO and kidney prognosis)* section.
Main results	16	(*a*) Give unadjusted estimates and, if applicable, confounder-adjusted estimates and their precision (eg, 95% confidence interval). Make clear which confounders were adjusted for and why they were included	Primary inference was prespecified to rely on multivariable Cox models. Unadjusted results were examined but not emphasized, as prespecified inference relied on multivariable models.
		(*b*) Report category boundaries when continuous variables were categorized	Category boundaries (quartiles of urinary mCHO, UPCR strata) are stated

			in the *Methods (Statistical Analysis)* and table footnotes.
		(*c*) If relevant, consider translating estimates of relative risk into absolute risk for a meaningful time period	Absolute risks (incidence rates per 1,000 person-years) are presented in Table 2.
Other analyses	17	Report other analyses done—eg analyses of subgroups and interactions, and sensitivity analyses	Subgroup and sensitivity analyses (MAKE30, UPCR strata, low-proteinuria subgroup, CIF, Fine-gray, Time dependent ROC) are described in *Results (Subgroup analysis among low UPCR patients)* and *Supplementary Materials*.
**Discussion**			
Key results	18	Summarise key results with reference to study objectives	Key findings are summarized in the first paragraph of the *Discussion*, referencing study objectives.
Limitations	19	Discuss limitations of the study, taking into account sources of potential bias or imprecision. Discuss both direction and magnitude of any potential bias	Study limitations (single center, observational design, single-time mCHO measurement, potential residual confounding) are discussed in the *Discussion*.
Interpretation	20	Give a cautious overall interpretation of results considering objectives, limitations, multiplicity of analyses, results from similar studies, and other relevant evidence	Overall interpretation considering objectives, limitations, and prior studies is provided in the *Discussion* section.
Generalisability	21	Discuss the generalisability (external validity) of the study results	External validity discussed in the Discussion, noting broad CKD population including both glomerular and non-glomerular etiologies
**Other information**			
Funding	22	Give the source of funding and the role of the funders for the present study and, if applicable, for the original study on which the present article is based	Funding sources (Sysmex Corporation and Aichi Kidney Foundation) and roles are described in the *Funding* and *Acknowledgements* sections.

*Give information separately for exposed and unexposed groups.

Note: An Explanation and Elaboration article discusses each checklist item and gives methodological background and published examples of transparent reporting. The STROBE checklist is best used in conjunction with this article (freely available on the Web sites of PLoS Medicine at http://www.plosmedicine.org/, Annals of

Internal Medicine at http://www.annals.org/, and Epidemiology at http://www.epidem.com/). Information on the STROBE Initiative is available at http://www.strobe-statement.org.
